# Dynamical behavior analysis of 2-control strategies on tuberculosis model

**DOI:** 10.1371/journal.pgph.0005875

**Published:** 2026-06-08

**Authors:** M. Abu Salek, Jannatun Nayeem, Muhammad Sajjad Hossain, M. Humayun Kabir

**Affiliations:** 1 Department of Mathematics, Jahangirnagar University, Dhaka, Bangladesh; 2 Mathematical and Computation Biology (MCB) Research Group, Jahangirnagar University, Dhaka, Bangladesh; 3 Department of Arts and Sciences, Ahsanullah University of Science and Technology, Dhaka, Bangladesh; Islamic Azad University South Tehran Branch, IRAN, ISLAMIC REPUBLIC OF

## Abstract

We develop and rigorously analyze a deterministic SEITR (Susceptible–Exposed–Infectious–Treated–Recovered) model that incorporates distancing and treatment interventions to examine the transmission dynamics of tuberculosis. The basic reproduction number (*R*_0_) is computed and decomposed to determine the threshold conditions for disease persistence. We propose a next-generation matrix formulation to determine the stability of the disease-free state and demonstrate that tuberculosis dies out whenever *R*_0_ < 1. Numerical implementation is performed using the fourth-order Runge–Kutta scheme and the backward sweep method, while Pontryagin’s maximum principle is utilized for determination of optimal intervention profiles. Sensitivity analysis is performed using normalized forward sensitivity indices, Pearson correlation, and Partial Rank Correlation Coefficient (PRCC). The results indicate that the transmission rate (β) is strongly positively correlated with the basic reproduction number (*R*_0_), whereas the control parameters (*u*_1_, *u*_2_) exhibit negative correlations with *R*_0_, highlighting their effectiveness in reducing disease transmission. The epidemiological efficacy of dual interventions is demonstrated by a significant decrease in infectious prevalence when both control measures are implemented simultaneously. Incremental cost-effectiveness ratio demonstrate that the combined control strategy is more cost-effective than individual interventions. These integrated analytical and numerical findings support the recommendation for sustained, early implementation of both distancing and treatment measures to achieve economically viable and dynamically stable tuberculosis mitigation.

## 1. Introduction

Tuberculosis (TB), caused by the bacterium Mycobacterium tuberculosis (MTB), is a major global health issue that mainly affects the lungs [1]. While lung infections are most common, the bacteria can spread to other organs through the bloodstream, leading to extra-pulmonary TB, which is usually non-infectious [2,3]. Active TB shows symptoms like a long-lasting cough, chest pain, fever, weight loss, and night sweats. It spreads through airborne droplets. Healthcare professionals diagnose tuberculosis (TB) using skin tests (TST) for latent tuberculosis infection, not active disease. Diagnosis typically occurs a few weeks after exposure, even if symptoms may not develop until later. Researchers employ an SEITR compartmental model to comprehend the transmission of tuberculosis. This aids in the formulation of effective and efficient public health strategies [4,5]. Understanding how stable and sensitive a person is helps to decide where to put treatment and social distancing efforts. This can help communities control TB better in the long run [6,7]. A lot of the current TB models aren’t very accurate because they don’t take into account things like seasonal changes, social awareness, or the best ways to treat the disease [4,8]. Despite advances in chemotherapy and vaccination, sustained transmission persists due to delayed diagnosis, incomplete treatment adherence, close contact to infected individuals due to lack of precautions, and socioeconomic determinants. Mathematical modeling has therefore been extensively employed to understand TB transmission mechanisms and to evaluate the effectiveness of intervention strategies [9]. Compartmental epidemiological models such as SEIR, SEIRS, SEITR, and fractional-order TB models have been widely investigated in different previous studies [4,6,10–13]. Stability analyses have traditionally focused on determining the basic reproduction number *R*_0_, whose threshold behavior distinguishes disease elimination from persistence [14]. Optimal control strategies incorporating treatment, vaccination, awareness, and behavioral modifications have been studied using Pontryagin’s Maximum Principle (PMP) [15,16]. Optimal control theory is a useful tool for finding ways to reduce disease while making the best use of available resources [5,17]. This method is especially helpful for keeping TB under control in places as per rare health care resources. By systematically simulating disease progression under various conditions, we can pinpoint critical transmission drivers and assess the sensitivity of interventions. Mathematical modeling has also been useful for figuring out how effective and cost-effective TB control programs will be in the long run. Additionally, behavioral factors and longer treatment needs are included to improve realism and lower the chances of disease reactivation [7,18].

However, existing tuberculosis models have largely treated stability analysis, sensitivity analysis, and optimal control as separate components, which limits a full understanding of disease dynamics. In this study, we develop a unified SEITR model that integrates these elements within a single model, explicitly linking control-dependent stability analysis with sensitivity assessment to better capture tuberculosis transmission behavior [19,20]. The discussion is expanded to incorporate the epidemiological context of tuberculosis in Bangladesh, highlighting persistent transmission dynamics and associated public health challenges [21]. Furthermore, dominance relationships between competing control strategies, i.e., distancing versus treatment have not been rigorously decomposed at the level of threshold structure. Further studies have highlighted the need to combine vaccination, treatment, and preventive measures to meet bio-medical and economic goals [5,22].

The SEITR model is proposed by introducing clear exposed and treated compartments to better capture the progression of TB and its control dynamics, such as effects of vaccination and treatment on pandemic influenza in [23]. We integrate optimal control strategies to evaluate the performance of interventions in different epidemiological scenarios and to reduce the overall disease burden. Previous research has shown that TB elimination is possible when the basic reproduction number is below one; otherwise, persistence is expected [24,25]. It has been rarely analyzed the joint impact of distancing and treatment controls on tuberculosis transmission within a single mathematical model, leaving a gap in understanding their combined influence on disease dynamics. To address this limitation, a structured deterministic SEITR model is developed to examine tuberculosis transmission under these control measures and to provide quantitative insight relevant to public health decision-making. Through stability analysis, sensitivity evaluation by using forward sensitivity indices, Latin Hypercube Sampling, Pearson and Partial Rank Correlation Coefficients (PRCC), and the determination of key epidemiological thresholds, the study clarifies how these control strategies can influence the reduction of tuberculosis disease burden like as [26]. The proposed model adds to the existing literature by offering a structured mathematical basis for assessing effective and sustainable TB control strategies. The following are the key components of this study:

a. In what ways can stability analysis identify the threshold conditions that naturally suppress the spread of tuberculosis within a population?
b. When treatment and distancing techniques are adjusted, which epidemiological parameters have the most impact on the dynamics of tuberculosis?
c. How much can the prioritization of scarce public health resources in tuberculosis control programs be influenced by sensitivity analysis?
d. How can the SEITR optimum control model’s cost-effectiveness evidence help inform national tuberculosis policy decisions to priorities controls with the greatest public health impact within budget?
e. Is it possible for mathematically optimized control systems to achieve sustainable tuberculosis reduction more effectively than traditional intervention approaches?

The study demonstrates how these issues are resolved by a mathematically rigorous SEITR model that includes stability, sensitivity, and optimal control evaluations. The results are presented to facilitate evidence-based decision-making for effective and sustainable tuberculosis prevention.

The rest of this article is organized by the following sections: Section 2 outlines the construction of the controlling SEITR model, including its basic assumptions and mathematical structure; Section 3 describes the materials and methods used for doing analytical work; Section 4 looks into how stable the disease-free and endemic equilibria are; Section 5 establishes the objective functional and determines the optimal control conditions; Section 6 does the sensitivity analysis to find the important parameters. Numerical simulations and the implications of the results for epidemiology are presented in Section 7; Section 8 assesses the model validation using available data; Section 9 examines the cost-effectiveness of the proposed intervention options; and finally, a summary of the key findings and a direction for future research are mentioned in Section 10.

## 2. Model formulation

**Fig 1:**
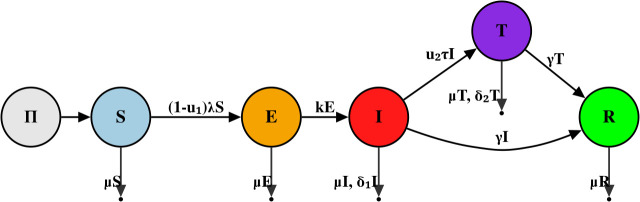
Diagram of TB transmission with control strategies.

The SEITR model is a compartmental approach that shows susceptible (*S*) people who are healthy but at risk of getting TB, exposed (*E*) people who have been exposed to TB but are not yet infectious, infectious (*I*) people who have active TB and can spread the disease to others, treated (*T*) people who are getting treatment for TB, and recovered (*R*) people, as shown in Fig 1. We integrate control variables into the SEITR model to accommodate actual measures designed to reduce the transmission of tuberculosis (TB). Optimal control theory allows us to identify the most effective strategies for minimizing both the number of infectious individuals and the costs associated with these interventions. Here, we define two key control variables such as Distance Control (*u*_1_(*t*)) and Treatment Control (*u*_2_(*t*)) in this model as [27]. The non-linear differential equations, which reflect the dynamics between compartments of ordinary differential equations as in [28], are given below. The description of the model parameters is provided in S1 Appendix [29].

The SEITR tuberculosis model with distancing control *u*_1_(*t*) and treatment control *u*_2_(*t*) has the following form


$$\begin{array}{c} \begin{array}{cc} \frac{dS}{dt} & =\Pi -(1-u_{1}(t))\;\lambda S-\mu S,\\ \frac{dE}{dt} & =(1-u_{1}(t))\;\lambda S-(\kappa +\mu )E,\\ \frac{dI}{dt} & =\kappa E-(\gamma +u_{2}(t)\tau +\mu +\delta_{1})I,\\ \frac{dT}{dt} & =u_{2}(t)\tau I-(\gamma +\mu +\delta_{2})T,\\ \frac{dR}{dt} & =\gamma I+\gamma T-\mu R, \end{array}\;\lambda =\frac{\beta I}{N},\;N=S+E+I+T+R. \end{array}$$ (1)

Throughout the stability analysis, it is assumed that the controls are admissible, i.e.,


$$\begin{array}{c} u_{1}(t),\;u_{2}(t)\in [0,1],\;\text{for all }t\geq 0, \end{array}$$

and the solutions are uniformly bounded in the positively invariant region


$$\begin{array}{c} \Gamma =\left\{(S,E,I,T,R)\in {\mathbb{R}}_{+}^{5}:\;N\leq \frac{\Pi }{\mu }\right\}, \end{array}$$

and continuously independent on the non-negative initial conditions $S_{0}\geq 0,E_{0}\geq 0,I_{0}\geq 0,T_{0}\geq 0,R_{0}\geq 0$. Therefore, the model (1) is biologically well-proposed.

Here, *u*_1_(*t*) is the distancing control rate and the coefficient $1-u_{1}(t)$ represents the prevention efforts to protect people in susceptible compartment from contacting infected individuals. And *u*_2_(*t*) represents the enhancement *t*reatment effort for individuals in the infectious compartment.

## 3. Mathematical analysis

### 3.1. Positivity and boundedness of the model

The given model equations are


$$\begin{array}{c} \begin{array}{c} \dot{S}=\Pi \left(1-u_{1}\right)\lambda S\geq -\left((1-u_{1})\lambda +\mu \right)S,\;\Rightarrow S(t)\geq S(0)e^{-\int_{0}^{t}((1-u_{1})\lambda +\mu )ds}\Rightarrow S(t)>0,\\ \dot{E}=\left(1-u_{1}\right)\lambda S-k_{1}E\geq -k_{1}E\Rightarrow E(t)\geq E(0)e^{-k_{1}t}\Rightarrow E(t)\geq 0,\\ \dot{I}=\kappa E-k_{2}I\geq -k_{2}I\Rightarrow I(t)\geq I(0)e^{-k_{2}t}\Rightarrow I(t)\geq 0,\\ \dot{T}=u_{2}I-k_{3}T\geq -k_{3}T\Rightarrow T(t)\geq T(0)e^{-k_{3}t}\Rightarrow T(t)\geq 0,\\ \dot{R}=\gamma I+\gamma T-\mu R\geq -\mu R\Rightarrow R(t)\geq R(0)e^{-\mu t}\Rightarrow R(t)\geq 0. \end{array} \end{array}$$

Since, *N* = *S* + *E* + *I* + *T* + *R*, then from (1) we have,


$$\begin{array}{c} \dot{N}=\Pi -\mu N-\delta I-\delta T\leq \Pi -\mu N \end{array}$$

Now,


$$\begin{array}{c} \dot{N}+\mu N\leq \Pi \Rightarrow N(t)\leq e^{-\mu t}N(0)+\frac{\Pi }{\mu }\left(1-e^{-\mu t}\right),\;\text{Hence,}\;\underset{t\to \infty }{lim sup}N(t)\leq \frac{\Pi }{\mu }. \end{array}$$

**Theorem 1** *The solution of the system is positively bounded for all* $\left(S(0),E(0),I(0),T(0),R(0)\right)\in R_{+}^{5}$ *and also define for the positive value of time t.*

### 3.2. Disease Free Equilibrium (DFE) and Basic Reproduction Number of the model

The model (1) exhibits a DFE, which is established by equating the right-hand sides of its equations to zero [30].


$$\begin{array}{c} E_{0}=\left(S,E,I,T,R\right)=\left(S^{*},0,0,0,0\right)=\left(\frac{\Pi }{\mu },0,0,0,0\right) \end{array}$$ (2)

The stability of the DFE, *E*_0_ will be analyzed by next-generation method [31]. Presented below are the non-negative matrix *P*, which denotes the new infection terms, and *V* is the non-singular *M*-matrix, which relates to the remaining transfer terms.


$$\begin{array}{cc} P & =[\begin{array}{cccc} 0 & \frac{(1-u_{1})\beta \Pi }{\mu } & 0 & 0\\ 0 & 0 & 0 & 0\\ 0 & 0 & 0 & 0\\ 0 & 0 & 0 & 0 \end{array}], \end{array}\begin{array}{cc} V & =[\begin{array}{cccc} k_{1} & 0 & 0 & 0\\ -\kappa & k_{2} & 0 & 0\\ 0 & -\tau u_{2} & k_{3} & 0\\ 0 & -\gamma & -\gamma & \mu \end{array}] \end{array}$$

$R_{0}=\rho (PV^{-1})$, where ρ signifies the spectral radius (the largest eigenvalue in absolute value) of the next generation matrix *PV*^−1^. Consequently, it can be inferred that


$$\begin{array}{cc} R_{0} & =\frac{(1-u_{1})\beta \Pi \kappa }{\mu k_{1}k_{2}} \end{array}$$

where, $k_{1}=\kappa +\mu ,k_{2}=\gamma +u_{2}\tau +\mu +\delta_{1},k_{3}=\gamma +\mu +\delta_{2}$.

### 3.3. Existence of the Endemic Equilibrium (EE)

**Theorem 2** *An endemic equilibrium E*_*1*_ *exists in* Γ *if and only if R*_*0*_ *> 1.*

An endemic equilibrium $E_{1}=(S^{**},E^{**},I^{**},T^{**},R^{**})$ is defined by requiring I^**^ > 0. At equilibrium, the following relations are obtained from (1).

From $\dot{I}=0$,


$$\begin{array}{c} \kappa E^{**}=(\gamma +u_{2}\tau +\mu +\delta_{1})I^{**}\;\Rightarrow \;E^{**}=\frac{\gamma +u_{2}\tau +\mu +\delta_{1}}{\kappa }\;I^{**}. \end{array}$$ (3)

From $\dot{E}=0$,


$$\begin{array}{c} (1-u_{1})\lambda^{**}S^{**}=(\kappa +\mu )E^{**}. \end{array}$$ (4)

Substituting (3) into (4) and using $\lambda^{**}=\beta I^{**}/N^{**}$ gives


$$\begin{array}{c} (1-u_{1})\beta \frac{S^{**}}{N^{**}}=(\kappa +\mu )\frac{\gamma +u_{2}\tau +\mu +\delta_{1}}{\kappa }. \end{array}$$ (5)

Since $0<S^{**}/N^{**}<1$, (5) implies that a strictly positive equilibrium requires


$$\begin{array}{c} (1-u_{1})\beta \kappa >(\kappa +\mu )(\gamma +u_{2}\tau +\mu +\delta_{1})⟺R_{0}>1. \end{array}$$

## 4. Stability analysis of the equilibria

### 4.1. Local stability of the DFE

**Theorem 3** *Let E*_*0*_ *denote the disease-free equilibrium of the system (1). Then E*_*0*_ *is locally asymptotically stable if R*_*0*_ *< 1, and unstable if R*_*0*_ *> 1.*

To establish the local stability of DFE consider the infected subsystem (*E*,*I*), because new infections are generated through transitions into the exposed class via $(1-u_{1})\lambda S$. The linearized infected subsystem around *E*_0_ is given by


$$\begin{array}{c} (\begin{array}{c} \dot{E}\\ \dot{I} \end{array})=(\begin{array}{cc} -(\kappa +\mu ) & (1-u_{1})\beta \\ \kappa & -(\gamma +u_{2}\tau +\mu +\delta_{1}) \end{array})(\begin{array}{c} E\\ I \end{array}). \end{array}$$ (6)

The trace of the matrix in (6) is negative. The determinant is computed as


$$\begin{array}{c} \det=(\kappa +\mu )(\gamma +u_{2}\tau +\mu +\delta_{1})-(1-u_{1})\beta \kappa . \end{array}$$ (7)

By rearranging (7), it is obtained that


$$\begin{array}{c} \det>0⟺\frac{(1-u_{1})\beta \kappa }{\left(\kappa +\mu )(\gamma +u_{2}\tau +\mu +\delta_{1}\right)}<1⟺R_{0}<1. \end{array}$$ (8)

Hence, the eigenvalues of (6) were shown to lie strictly in the left half-plane when *R*_0_ < 1, establishing local asymptotic stability.

### 4.2. Global Stability of the DFE

**Theorem 4** *The disease-free equilibrium E*_*0*_ *is globally asymptotically stable in the positively invariant region*


$$\begin{array}{c} \Gamma =\left\{(S,E,I,T,R)\in {\mathbb{R}}_{+}^{5}:N\leq \frac{\Pi }{\mu }\right\} \end{array}$$

whenever *R*_0_ < 1.

To show global stability, the infected subsystem is bounded by a comparison system. Since S(t)≤N(t)≤Π/μ in Γ, it is obtained that


$$\begin{array}{c} (1-u_{1})\lambda S=(1-u_{1})\frac{\beta I}{N}S\leq (1-u_{1})\beta I. \end{array}$$

Hence the exposed equation satisfies


$$\begin{array}{c} \dot{E}\leq (1-u_{1})\beta I-(\kappa +\mu )E, \end{array}$$

and the infected equation remains as


$$\begin{array}{c} \dot{I}=\kappa E-(\gamma +u_{2}\tau +\mu +\delta_{1})I. \end{array}$$

Thus, (*E*,*I*) is dominated by the linear comparison system


$$\begin{array}{c} (\begin{array}{c} \dot{E}\\ \dot{I} \end{array})\leq A(\begin{array}{c} E\\ I \end{array}),\;A=(\begin{array}{cc} -(\kappa +\mu ) & (1-u_{1})\beta \\ \kappa & -(\gamma +u_{2}\tau +\mu +\delta_{1}) \end{array}). \end{array}$$

When *R*_0_ < 1, the matrix *A* is shown to be Hurwitz by (8), and therefore the comparison system decayed to zero. By the comparison principle, it is concluded that E(t)→0 and I(t)→0 as t→∞. Substituting into the remaining equations for *T* and *R* implies that T(t)→0 and R(t)→0, while S(t)→Π/μ.

### 4.3. Local stability of the Endemic Equilibrium (EE)

**Theorem 5** *The endemic equilibrium E*_*1*_ *is locally asymptotically stable whenever R*_*0*_ *> 1.*

Local stability is established by the Jacobian evaluated at *E*_1_. Since the model is five-dimensional, here we consider only the infected subsystem (*E*,*I*).

Let $x=(E,I{)}^{T}$. New infection terms appear only in the *E*-equation:


$$\begin{array}{c} F(E,I)=(\begin{array}{c} \frac{(1-u_{1})\beta SI}{N}\\ 0 \end{array}),\;V(E,I)=(\begin{array}{c} (\kappa +\mu )E\\ (\gamma +u_{2}\tau +\mu +\delta_{1})I-\kappa E \end{array}). \end{array}$$

We linearize around the equilibrium and let us consider S^**^ and N^**^ are fixed.

Thus near *E*_1_, the infection term is approximated by


$$\begin{array}{c} (1-u_{1})\beta \frac{SI}{N}\approx (1-u_{1})\beta \frac{S^{**}}{N^{**}}\;I. \end{array}$$

So the reduced Jacobian (infected block) is


$$\begin{array}{c} J_{I}(E_{1})=(\begin{array}{cc} -(\kappa +\mu ) & (1-u_{1})\beta \frac{S^{**}}{N^{**}}\\ \kappa & -(\gamma +u_{2}\tau +\mu +\delta_{1}) \end{array}). \end{array}$$

The characteristic polynomial is


$$\begin{array}{c} \det(\lambda I-J_{I})=|\begin{array}{cc} \lambda +\kappa +\mu & -(1-u_{1})\beta \frac{S^{**}}{N^{**}}\\ -\kappa & \lambda +\gamma +u_{2}\tau +\mu +\delta_{1} \end{array}|=(\lambda +\kappa +\mu )(\lambda +\gamma +u_{2}\tau +\mu +\delta_{1})-\kappa (1-u_{1})\beta \frac{S^{**}}{N^{**}}. \end{array}$$

Hence


$$\begin{array}{c} P(\lambda )=\lambda^{2}+(\kappa +\mu +\gamma +u_{2}\tau +\mu +\delta_{1})\lambda +\left((\kappa +\mu )(\gamma +u_{2}\tau +\mu +\delta_{1})-\kappa (1-u_{1})\beta \frac{S^{**}}{N^{**}}\right). \end{array}$$

For a second-order polynomial


$$\begin{array}{c} \lambda^{2}+\alpha_{1}\lambda +\alpha_{0}, \end{array}$$

local asymptotic stability is equivalent to:


$$\begin{array}{c} \alpha_{1}>0,\;\alpha_{0}>0. \end{array}$$

Here,


$$\begin{array}{c} \alpha_{1}=\kappa +\mu +\gamma +u_{2}\tau +\mu +\delta_{1}>0\;\text{automatically (all parameters positive).} \end{array}$$

Thus stability reduces to the sign of


$$\begin{array}{c} \alpha_{0}=(\kappa +\mu )(\gamma +u_{2}\tau +\mu +\delta_{1})-\kappa (1-u_{1})\beta \frac{S^{**}}{N^{**}}. \end{array}$$

Now from the endemic equilibrium derivation (5):


$$\begin{array}{c} (1-u_{1})\beta \frac{S^{**}}{N^{**}}=(\kappa +\mu )\frac{\gamma +u_{2}\tau +\mu +\delta_{1}}{\kappa }. \end{array}$$

Using the above condition into $\alpha_{0}$,


$$\begin{array}{c} \alpha_{0}=(\kappa +\mu )(\gamma +u_{2}\tau +\mu +\delta_{1})-\kappa (1-u_{1})\beta \frac{S^{**}}{N^{**}}=(\kappa +\mu )(\gamma +u_{2}\tau +\mu +\delta_{1})-(\kappa +\mu )(\gamma +u_{2}\tau +\mu +\delta_{1})=0. \end{array}$$

So at the endemic equilibrium, the reduced infected Jacobian has


$$\begin{array}{c} P(\lambda )=\lambda^{2}+(\kappa +\mu +\gamma +u_{2}\tau +\mu +\delta_{1})\lambda . \end{array}$$

Hence the eigenvalues are


$$\begin{array}{c} \lambda_{1}=0,\;\lambda_{2}=-(\kappa +\mu +\gamma +u_{2}\tau +\mu +\delta_{1})<0. \end{array}$$

The characteristic polynomial of the infected subsystem satisfies the Routh-Hurwitz conditions when *R*_0_ > 1.Thus, the endemic equilibrium is locally asymptotically stable whenever it exists (i.e., for *R*_0_ > 1).

### 4.4. Global stability of the Endemic Equilibrium (EE)

**Theorem 6** *Assume that the controls are constants* $u_{1},u_{2}\in [0,1]$ *and that R*_*0*_ *> 1 so that the endemic equilibrium* $E_{1}=(S^{**},E^{**},I^{**},T^{**},R^{**})$ *exists with* I^**^ > 0*. Then E*_*1*_ *is globally asymptotically stable in the positively invariant region* $\Gamma ={\mathbb{R}}_{+}^{5}$*.*

***Proof.*** Define the Lyapunov function on *{S,E,I > 0}:*


$$\begin{array}{c} V(S,E,I)=(S-S^{**}-S^{**}\ln\frac{S}{S^{**}})+c_{1}(E-E^{**}-E^{**}\ln\frac{E}{E^{**}})+c_{2}(I-I^{**}-I^{**}\ln\frac{I}{I^{**}}), \end{array}$$

where $c_{1},c_{2}>0$ will be chosen. Since x−1−lnx≥0 for *x* > 0, it follows that *V* ≥ 0 and *V* = 0 if and only if $S=S^{**}$, $E=E^{**}$, and *I* = I^**^. Differentiating along solutions gives


$$\begin{array}{c} \dot{V}=(1-\frac{S^{**}}{S})\dot{S}+c_{1}(1-\frac{E^{**}}{E})\dot{E}+c_{2}(1-\frac{I^{**}}{I})\dot{I}. \end{array}$$

Choose


$$\begin{array}{c} c_{1}=\frac{\kappa }{\kappa +\mu },\;c_{2}=\frac{\kappa }{\gamma +u_{2}\tau +\mu +\delta_{1}}. \end{array}$$

Using the equilibrium identity $\Pi =(1-u_{1})\lambda^{**}S^{**}+\mu S^{**}$ and the relations (3)-(5), $\dot{V}$ can be arranged as


$$\begin{array}{c} \dot{V}=-\mu \frac{(S-S^{**}{)}^{2}}{S}-(\kappa +\mu )c_{1}\;\frac{(E-E^{**}{)}^{2}}{E}-(\gamma +u_{2}\tau +\mu +\delta_{1})c_{2}\;\frac{(I-I^{**}{)}^{2}}{I}+\;(1-u_{1})\lambda^{**}S^{**}\;\Xi , \end{array}$$

where, after normalization


$$\begin{array}{c} x=\frac{S}{S^{**}},\;z=\frac{I}{I^{**}},\;w=\frac{N}{N^{**}}, \end{array}$$

the remaining term takes the form


$$\begin{array}{c} \Xi =3-\frac{1}{x}-\frac{xz}{w}-\frac{w}{z}. \end{array}$$

Let $A=\frac{1}{x}$, $B=\frac{xz}{w}$, and $C=\frac{w}{z}$. These are positive and satisfy *ABC* = 1. Hence, by arithmetic mean (AM) and geometric mean (GM),


$$\begin{array}{c} \frac{A+B+C}{3}\geq \sqrt[{3}]{ABC}=1\;\Rightarrow \;A+B+C\geq 3\;\Rightarrow \;\Xi \leq 0. \end{array}$$

Therefore $\dot{V}\leq 0$ for all $(S,E,I)\in {\mathbb{R}}_{+}^{3}$. Moreover, $\dot{V}=0$ holds only when $S=S^{**}$, $E=E^{**}$, *I* = I^**^ and simultaneously *A* = *B* = *C* = 1, which yields $N=N^{**}$. On this invariant set, the remaining linear subsystem


$$\begin{array}{c} \dot{T}+(\gamma +\mu +\delta_{2})T=u_{2}\tau I,\;\dot{R}+\mu R=\gamma I+\gamma T \end{array}$$

implies $T(t)\to T^{**}$ and $R(t)\to R^{**}$. By LaSalle’s invariance principle, every trajectory in Γ converges to *E*_1_. Thus *E*_1_ is globally asymptotically stable.

### 4.5. Absence of backward bifurcation

**Lemma 1** *Consider the SEITR system (1) with standard incidence* λ=βI/N *and constant controls* $u_{1},u_{2}\in [0,1]$*. If* $R_{0}=\frac{(1-u_{1})\beta \kappa }{\left(\kappa +\mu )(\gamma +u_{2}\tau +\mu +\delta_{1}\right)}$*.*

*Then the following statements hold:*

(i) *The disease-free equilibrium exists for all parameter values and is locally asymptotically stable whenever R*_*0*_ *< 1.*
(ii) *A strictly positive endemic equilibrium exists only when R*_*0*_ *> 1 and is unique in the feasible region.*

*Consequently, no endemic equilibrium can coexist with the disease-free equilibrium for R*_*0*_ *< 1, and therefore a backward bifurcation at R*_*0*_ *= 1 does not occur; the system undergoes a forward (transcritical) bifurcation at R*_*0*_ *= 1.*

## 5. Objective functional of optimal control

The objective functional quantifies the trade-off between reducing the infectious population and minimizing intervention costs over a fixed time horizon. It is expressed as:


$$\begin{array}{c} minJ(u_{1},u_{2})=\int_{0}^{T}[a_{1}E+a_{2}I+a_{3}T+\frac{1}{2}(a_{4}u_{1}^{2}+a_{5}u_{2}^{2})]dt. \end{array}$$

where, *a*_1_, *a*_2_, *a*_3_, *a*_4_ and *a*_5_ are weight parameters and all are positive. The squared terms represent the cost of control measures, assuming they increase quadratically with their intensity.

### 5.1. Characterization of the optimal control

By Pontryagin’s Maximum Principle (PMP), which transforms the problem into a system of differential equations involving state variables *S*(*t*), *E*(*t*), *I*(*t*), *T*(*t*) and *R*(*t*) adjoint variables: $\Lambda_{S},\Lambda_{E},\Lambda_{I},\Lambda_{T}$ and $\Lambda_{R}$ and Hamiltonian. The Hamiltonian incorporates the objective function and state dynamics as:


$$\begin{array}{cc} H & =a_{1}E+a_{2}I+a_{3}T+\frac{a_{4}u_{1}^{2}}{2}+\frac{a_{5}u_{2}^{2}}{2}+\Lambda_{S}[\Pi -(1-u_{1})\lambda S-\mu S]+\Lambda_{E}[(1-u_{1})\Lambda S-\kappa E-\mu E]\\ & +\Lambda_{I}[\kappa E-\gamma I-u_{2}\tau I-\mu I-\delta_{1}I]+\Lambda_{T}[u_{2}\tau I-\gamma T-\mu T-\delta_{2}T]+\Lambda_{R}[\gamma I+\gamma T-\mu R] \end{array}$$

where, $\Lambda_{S},\Lambda_{E},\Lambda_{I},\Lambda_{T}$ and $\Lambda_{R}$ are adjoint variables.


$$\begin{array}{c} \begin{array}{cc} \Lambda_{S}^{{\;}^{\prime }} & =(1-u_{1})\lambda \Lambda_{S}+\mu \Lambda_{S}-(1-u_{1})\lambda \Lambda_{E}\\ \Lambda_{E}^{{\;}^{\prime }} & =-a_{1}+\kappa \Lambda_{E}+\mu \Lambda_{E}-\kappa \Lambda_{I}\\ \Lambda_{I}^{{\;}^{\prime }} & =-a_{2}+\gamma \Lambda_{I}+u_{2}\tau \Lambda_{I}+\mu \Lambda_{I}+\delta_{1}\Lambda_{I}-u_{2}\tau \Lambda_{T}-\gamma \Lambda_{R}\\ \Lambda_{T}^{{\;}^{\prime }} & =-a_{3}+\gamma \Lambda_{T}+\mu \Lambda_{T}+\delta_{2}\Lambda_{T}-\gamma \Lambda_{R}\\ \Lambda_{R}^{{\;}^{\prime }} & =\mu \Lambda_{R} \end{array} \end{array}$$

The adjoint variables $\Lambda_{i}$ satisfying $\frac{d\Lambda_{i}}{dt}=\frac{dH}{dt}$ with the transversity conditions, $\Lambda_{i}(T)=0$, *i* = *S*, *E*, *I*, *T*, *R*.

The optimality condition is given by $\frac{dH}{du_{i}}=0$, *i* = 1, 2.

For the control *u*_1_ we have, $\frac{H}{u_{1}}{|}_{u_{1}=u_{1}^{*}}=0$ $⟹a_{4}u_{1}^{*}+\lambda S\Lambda_{S}-\lambda S\Lambda_{E}=0$ $⟹u_{1}^{*}=\frac{\beta IS(\Lambda_{E}-\Lambda_{S})}{a_{4}}$ and, for the control *u*_2_ we can write, $\frac{H}{u_{2}}{|}_{u_{2}=u_{2}^{*}}=0$ $⟹a_{5}u_{2}^{*}+\tau I\Lambda_{I}+\tau I\Lambda_{T}=0$


$$\begin{array}{c} ⟹u_{2}^{*}=\frac{\tau I(\Lambda_{I}-\Lambda_{T})}{a_{5}} \end{array}$$

So, we have the controls,

$u_{1}^{*}=min\left(1,max\left(0,\frac{\beta IS(\Lambda_{E}-\Lambda_{S})}{a_{4}}\right)\right)$ and $u_{2}^{*}=min\left(1,max\left(0,\frac{\tau I(\Lambda_{I}-\Lambda_{T})}{a_{5}}\right)\right).$

## 6. Sensitivity analysis

Sensitivity analysis is a quantitative tool for determining how differences in model parameters affect the behaviour or consequences of a mathematical system. A global sensitivity analysis is performed using Latin Hypercube Sampling (LHS) combined with standardized regression coefficients (SRC), Pearson correlation coefficients, and Partial Rank Correlation Coefficients (PRCC)(S1 Fig). A total of *N* = 1000 parameter sets are generated within biologically feasible ranges. The basic reproduction number *R*_0_ is evaluated for each sampled set and statistical associations between parameters and *R*_0_ are quantified.

### 6.1. Analytical analysis of sensitive parameters

To analyze the sensitivity we defined an explicit formula for *R*_0_ as,


$$\begin{array}{c} \chi_{r}^{R_{0}}=\left(\frac{r}{R_{0}}\right)\left(\frac{\partial R_{0}}{\partial r}\right) \end{array}$$

where, $R_{0}=R_{0}(\beta ,\kappa ,\gamma ,\tau ,\delta_{1},u_{1},u_{2})$ and *r* represents a parameter. Certain key parameters of the model (1) have a significant impact on the basic reproduction number, *R*_0_. The numerical expressions for the respective parameters of the model are defined as follows:


$$\begin{array}{c} \begin{array}{cc} \frac{\partial R_{0}}{\partial \beta } & =\frac{\Pi \kappa (1-u_{1})}{\mu (\kappa +\mu )(\gamma +u_{2}\tau +\mu +\delta_{1})}\\ \frac{\partial R_{0}}{\partial \kappa } & =\frac{\beta \Pi (1-u_{1})}{\mu (\kappa +\mu {)}^{2}}\\ \frac{\partial R_{0}}{\partial \gamma } & =-\frac{\beta \Pi \kappa (1-u_{1})}{\mu (\kappa +\mu )(\gamma +u_{2}\tau +\mu +\delta_{1}{)}^{2}}\\ \frac{\partial R_{0}}{\partial \tau } & =-\frac{\beta \Pi \kappa (1-u_{1})u_{2}}{\mu (\kappa +\mu )(\gamma +u_{2}\tau +\mu +\delta_{1}{)}^{2}}\\ \frac{\partial R_{0}}{\partial \delta_{1}} & =-\frac{\beta \Pi \kappa (1-u_{1})}{\mu (\kappa +\mu )(\gamma +u_{2}\tau +\mu +\delta_{1}{)}^{2}}\\ \frac{\partial R_{0}}{\partial u_{1}} & =-\frac{\beta \Pi \kappa }{\mu (\kappa +\mu )(\gamma +u_{2}\tau +\mu +\delta_{1})}\\ \frac{\partial R_{0}}{\partial u_{2}} & =-\frac{\beta \Pi \kappa (1-u_{1})\tau }{\mu (\kappa +\mu )(\gamma +u_{2}\tau +\mu +\delta_{1}{)}^{2}} \end{array} \end{array}$$ (9)

**Algorithm 1 Stepwise procedure for computing the optimal controls $u_{1}^{*}(t)$ and $u_{2}^{*}(t)$ in the controlled SEITR model**

**Input:**  Model parameters $\Pi ,\beta ,\kappa ,\gamma ,\tau ,\mu ,\delta_{1},\delta_{2}$; weights $a_{1},a_{2},a_{3},a_{4},a_{5}>0$; time horizon [0,*T*]; initial states *S*(0),*E*(0),*I*(0),*T*(0),*R*(0); tolerance ε>0; maximum iterations *M*.

**Output:** Optimal control profiles $u_{1}^{*}(t),u_{2}^{*}(t)$ and optimal state trajectories $S^{*}(t),E^{*}(t),I^{*}(t),T^{*}(t),R^{*}(t)$.

**Step 1 (Initialize).** Set an initial guess on [0,*T*], e.g., $u_{1}^{\left(0\right)}(t)\equiv 0$, $u_{2}^{\left(0\right)}(t)\equiv 0$, and set *m* = 0.

**Step 2 (Forward state solve).** With $u_{1}^{\left(m\right)}(t)$ and $u_{2}^{\left(m\right)}(t)$, solve the SEITR state system forward on [0,*T*] to obtain $\left(S^{\left(m\right)}(t),E^{\left(m\right)}(t),I^{\left(m\right)}(t),T^{\left(m\right)}(t),R^{\left(m\right)}(t)\right)$. Compute $\lambda^{\left(m\right)}(t)=\frac{\beta I^{\left(m\right)}(t)}{N^{\left(m\right)}(t)}$, where $N^{\left(m\right)}(t)=S^{\left(m\right)}(t)+E^{\left(m\right)}(t)+I^{\left(m\right)}(t)+T^{\left(m\right)}(t)+R^{\left(m\right)}(t)$.

**Step 3 (Terminal conditions).** Impose $\Lambda_{S}(T)=\Lambda_{E}(T)=\Lambda_{I}(T)=\Lambda_{T}(T)=\Lambda_{R}(T)=0$.

**Step 4 (Backward adjoint solve).** Using the trajectories from Step 2, solve the adjoint system backward on [0,*T*]:

$\Lambda_{S}^{\prime }(t)=(1-u_{1}^{\left(m\right)}(t))\lambda^{\left(m\right)}(t)\Lambda_{S}(t)+\mu \Lambda_{S}(t)-(1-u_{1}^{\left(m\right)}(t))\lambda^{\left(m\right)}(t)\Lambda_{E}(t),\;\Lambda_{E}^{\prime }(t)=-a_{1}+(\kappa +\mu )\Lambda_{E}(t)-\kappa \Lambda_{I}(t),$ $\Lambda_{I}^{\prime }(t)=-a_{2}+(\gamma +u_{2}^{\left(m\right)}(t)\tau +\mu +\delta_{1})\Lambda_{I}(t)-u_{2}^{\left(m\right)}(t)\tau \Lambda_{T}(t)-\gamma \Lambda_{R}(t),\;\Lambda_{T}^{\prime }(t)=-a_{3}+(\gamma +\mu +\delta_{2})\Lambda_{T}(t)-\gamma \Lambda_{R}(t),\;\Lambda_{R}^{\prime }(t)=\mu \Lambda_{R}(t).$

**Step 5 (Control update).** Update pointwise and project onto [0,1]:

$u_{1}^{\left(m+1\right)}(t)=\min\;\left\{1,\max\;\left(0,\;\frac{\beta \;I^{\left(m\right)}(t)\;S^{\left(m\right)}(t)(\Lambda_{E}(t)-\Lambda_{S}(t))}{a_{4}\;N^{\left(m\right)}(t)}\right)\right\},$

$u_{2}^{\left(m+1\right)}(t)=\min\;\left\{1,\max\;\left(0,\;\frac{\tau \;I^{\left(m\right)}(t)(\Lambda_{I}(t)-\Lambda_{T}(t))}{a_{5}}\right)\right\}.$

**Step 6 (Convergence check).** If $\|u_{1}^{\left(m+1\right)}-u_{1}^{\left(m\right)}{\|}_{\infty }+\|u_{2}^{\left(m+1\right)}-u_{2}^{\left(m\right)}{\|}_{\infty }<\varepsilon$, stop and set $u_{i}^{*}(t)=u_{i}^{\left(m+1\right)}(t)$ for *i* = 1,2.

**Step 7 (Iterate).** Otherwise set m←m+1; if *m* < *M*, return to Step 2, else stop and report the last iterate as the numerical approximation.

After analytic simplification considering (9), the results are summarized in S1 Table, using the above explicit derivative formula in relation to the model parameters, based on the values provided in S1 Appendix.

### 6.2. Statistical significance

The statistical significance of PRCC values was evaluated using the test statistic


$$\begin{array}{c} t=\frac{r\sqrt{N-k-2}}{\sqrt{1-r^{2}}} \end{array}$$ (10)

with df=N−k−2 degrees of freedom. For *N* = 1000 and *k* = 6, *df* = 992. The factors *u*_1_, β, $\delta_{1}$, and γ are determined to be highly statistically significant (*p* < 0.001), suggesting that their effect on *R*_0_ is improbable to result from random sampling variability. The remaining parameters displayed weak correlations with relatively high p-values, indicating minimal statistical influence on *R*_0_ within the examined ranges. Due to PRCC values approaching ±0.95 with N = 1000 resulting in exceedingly huge t-statistics, the p-values are essentially < 0.001 (in fact, significantly less).

**Fig 2:**
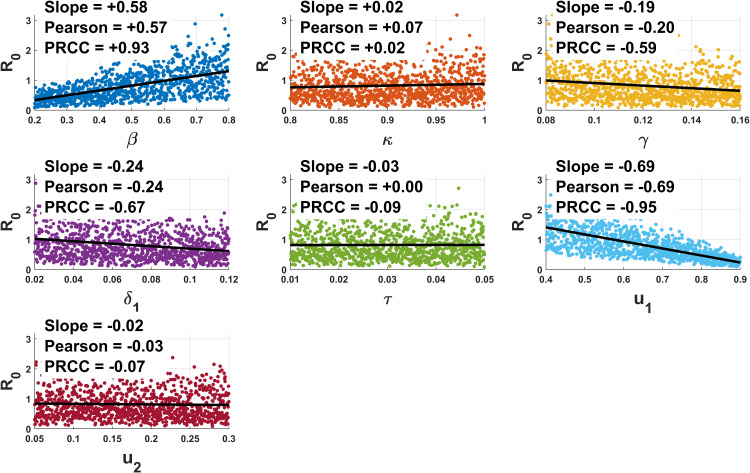
Regression, Pearson and PRCC analysis of model parameters by scatter diagram.

The scatter plots in Fig 2 (S1 Fig) show the correlations between each model parameter and the basic reproduction number *R*_0_, with Slope, Pearson, and PRCC values in each panel. Increased contact reduction significantly reduces transmission potential, as shown by a major negative influence on the distance control parameter *u*_1_ (Slope = -0.68674, Pearson = -0.69009, PRCC = -0.95354), with a 95% confidence interval (-0.95885, -0.94756). The transmission rate β is positively correlated with epidemic intensity (Slope = 0.58425, Pearson = 0.57036, PRCC = 0.93028, 95% confidence limits (0.92143, 0.93817), indicating extreme sensitivity to transmission efficiency variations. Shortening the infectious time has moderate negative impacts on the disease-induced death rate $\delta_{1}$ (PRCC = -0.66736) and recovery rate γ (PRCC = -0.59017), highlighting its epidemiological significance. The progression rate κ (PRCC = 0.023958), treatment rate τ (PRCC = -0.087536), and treatment control *u*_2_ (PRCC = -0.072582) have weak sensitivities, indicating limited impact in the examined parameter ranges. Slope and Pearson values are consistent, confirming linear trends, while PRCC magnitudes imply monotonic correlations after correcting for parameter interactions. The figure shows that contact reduction and transmission intensity dominate transmission dynamics, while treatment-related processes have a smaller impact on *R*_0_. The statistical significance and the sensitivity interpretation of *R*_0_ of the influential parameters of the model are described in the following S2 Table.

In this study, both Standardized regression coefficients (SRC) and Partial Rank Correlation Coefficient (PRCC) recognised *u*_1_ and β as dominating parameters; however, PRCC values had stronger magnitudes. This shows that monotonic correlations exist, and that PRCC provides a more reliable assessment of parameter influence in nonlinear epidemiological systems. As a result, PRCC is regarded as the most trustworthy sensitivity metric for assessing model behaviour.

Overall, the table shows that epidemic transmission is primarily governed by mechanisms controlling effective contact and transmission intensity, while removal and treatment processes have a secondary influence, and disease progression plays only a minor role in determining *R*_0_ within the specified parameter range. Finally, the Fig 2 depicts the impact of parameters, while the statistical formula (10) verifies the statistical significance of that impact.

**Fig 3:**
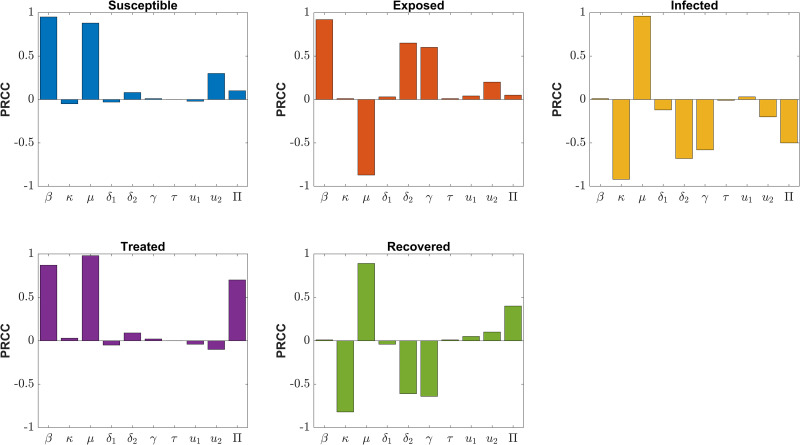
PRCC indices of all parameters for each compartments of the model.

Fig 3 shows the Partial Rank Correlation Coefficient (PRCC) values for various parameters impacting the five compartmental model with distance and treatment controls. The bar graphs illustrate the sensitivity of each compartment to changes in parameters such as transmission rate (β), progression rate (κ), recovery rate (γ), death rates (μ, $\delta_{1}$, $\delta_{2}$), control methods (*u*_1_, *u*_2_), and recruitment rate (Π). Parameters with bars close to 1 or -1 have the most impact. If the value is positive, increasing the parameter raises the number of members in that group. If the value is negative, reducing the parameter reduces the number.

From an epidemiological approach, the PRCC analysis identifies the parameters with the most influence on the model’s compartments. A strong positive PRCC value for transmission rate β and progression rate κ in susceptible and exposed populations implies that these parameters significantly influence individuals’ transition into infection. Distancing control *u*_1_, treatment control *u*_2_, and disease-related death rates all have substantial negative relationships with the infected class, showing their effectiveness in reducing active infection. Treatment effort *u*_2_ and recovery rate γ have significant effects on treated and recovered compartments, highlighting the need of maximizing contact reduction, treatment efficiency, and recovery in tuberculosis control.

### 6.3. Sensitivity analysis of different parameters

The sensitivity analysis presented in the figures highlights the differential impact of varying parameters β, γ, and κ on the infectious population over 50 years.

**Fig 4:**
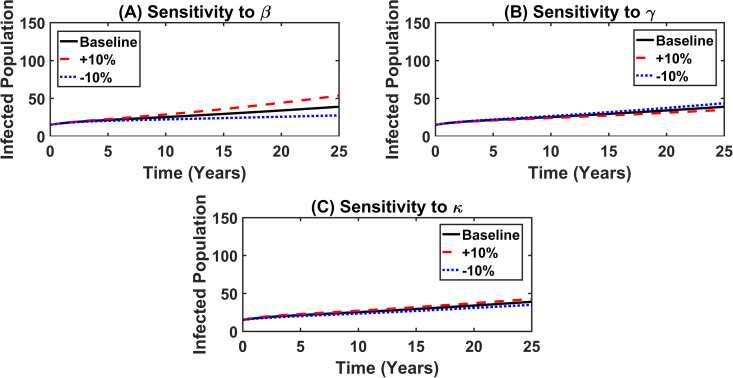
Sensitivity analysis impact on infectiousness for β, γ, and κ.

In Fig 4 (S1 Fig), the parameter β (Fig 4A), likely representing the effective contact rate, exhibits a substantial increase in the infectious population with a + 10% elevation (dashed red line) relative to the baseline (solid black line). A −10% reduction (dotted blue line) demonstrates the opposite effect, indicating that the population is very responsive to fluctuations in transmission. In Fig 4B, the parameter γ illustrates the recovery rate; a + 10% rise (dashed red line) results in a reduction in the number of individuals who are ill, as a greater number of individuals recover. Conversely, a −10% reduction (dotted blue line) results in a significant rise in the number of individuals who are ill, highlighting the critical role of recovery in halting the transmission. In a similar way, for κ (Fig 4C), which presumably represents the transition rate from exposed to infectious, a + 10% increase (dashed red line) results in a heightened infectious population, whilst a −10% decrease (dotted blue line) yields a diminished one. Nonetheless, its effect is less significant than that of β or γ. These statistics indicate that the infectious population is highly responsive to variations in the effective contact rate (β) and recovery rate (γ), highlighting these parameters as essential targets for intervention efforts to manage the disease.

## 7. Model validation

The data on tuberculosis cases in Bangladesh shown in the picture are from the World Health Organization’s Global Tuberculosis Report database and the World Bank’s World Development Indicators repository [32] (S1 Data). For curve fitting and projection, a nonlinear polynomial regression method is used, and the least-squares optimization method is employed to obtain the model coefficients. The coefficient of determination (R²), which is greater than 0.97, is used to assess how well the fitted model performs. This means that the observed and modeled values are quite close.

**Fig 5:**
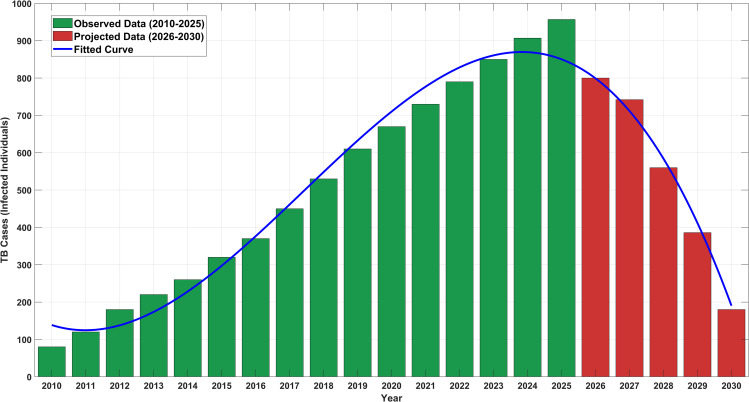
Curve fitting of infected cases with model prediction with projection up to 2030.

In Fig 5 (S1 Fig), TB Case Projection with Fitted Curve (2010–2030) visually represents. In this figure, the green bars correspond to the observed TB case data from 2010 to 2025 [33], based on surveillance and reporting. The red bars project TB cases from 2026 to 2030 using the SEITR model, which incorporates disease progression and control strategies such as treatment and social distancing. A smooth blue curve has been fitted across the entire data set using nonlinear regression or similar techniques to capture the trend of infection spread over time.

The curve fitting serves as a validation tool to assess how accurately the SEITR model captures the real behavior of TB transmission dynamics. The fitted curve shows that the number of TB cases steadily rises until it reaches a peak around 2025, after which it slowly falls during the forecast period. This pattern demonstrates how actions modelled in the system, such as increasing treatment rates and behavioural modifications like distance, have an impact. The fact that the curve and the real data (green bars) are so similar during the years studied shows that the model is very stable, which means it will continue to be useful for tracking population-level TB changes over time. The model is also quite sensitive to control factors, as seen by the red bars showing a decrease in anticipated cases. This means that good treatment and distance can greatly lower the number of TB cases by 2030. This demonstrates that mathematical modelling not only improves understanding of disease patterns, but it also serves as a forecasting tool for policymakers.

**Fig 6:**
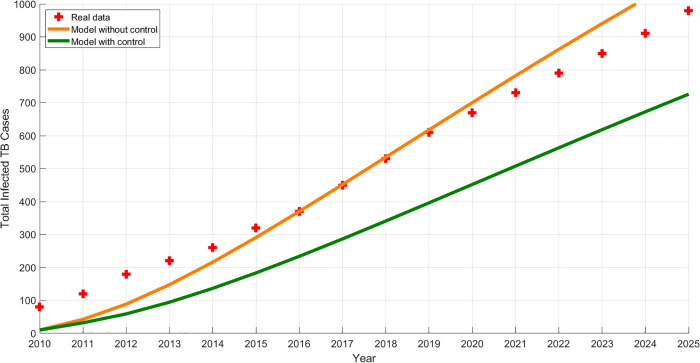
Model Fit to Real Data (2010-2025) of total TB cases assessed with and without control measures.

Fig 6 (S1 Fig) illustrates how model-generated trends from 2010 to 2025 compare to reported TB cases (S1 Text). The red plus signs depict real TB case data from 2010 to 2025, which shows a steady growth from fewer than 100 cases to almost 1000. The orange line, which shows the model without any controls, follows this pattern quite closely and keeps going up fast. This means that the model is correct when it comes to how TB spreads without any controls. The green line, which shows the model with controls (such treatment and distancing), goes up considerably more slowly and cuts down on the number of TB cases. The space between the green and orange lines clearly stops at about 700 cases in 2025. This slower rise shows that interventions are effective and how much control approaches may change things. Over time, timely measures can considerably reduce TB cases, as seen in the figure.

## 8. Results and discussions

### 8.1. Numerical technique

Three numerical methods are used to approximate the governing system: the fourth-order Runge–Kutta method for deterministic simulation, finite difference schemes for spatial discretisation, and implicit solvers for stiff regime stability. Step-size refinement and technique comparisons proved the accuracy and consistency of the calculated trajectories, preserving the epidemic dynamics’ qualitative behaviour across computing methodologies.

### 8.2 Comparisons for infectious and treatment population trajectories using control strategies

**Fig 7:**
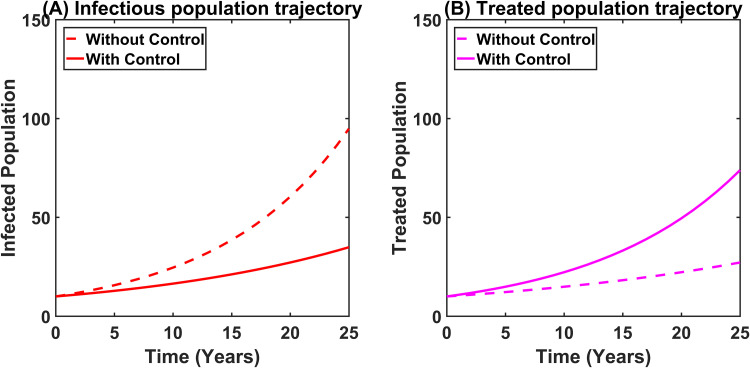
Comparison of infectious and treated population trajectories under controls.

The long-term effects of control strategies on infected and treated populations over a 25-year period are shown in Fig 7 (Fig 7A and Fig 7B (S1 Fig)). When compared to the uncontrolled situation, a significant decrease in infection levels is seen under controlled conditions, indicating the efficacy of the implemented interventions. By preventing the spread of disease, control techniques significantly reduce the strain on treatment resources, as seen by a corresponding drop in treatment demand.

**Fig 8:**
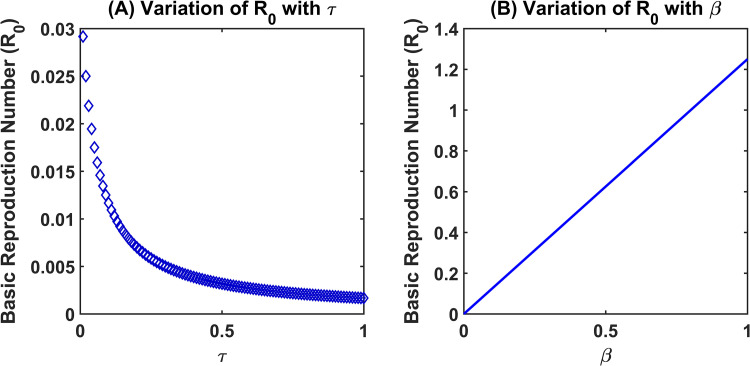
Sensitivity of the basic reproduction number (*R*_0_) with respect to parameters τ and β.

Fig 8 (Fig 8A and Fig 8B (S1 Fig)) illustrate how the basic reproduction number (*R*_0_) changes in response to variations in the treatment parameter τ and the transmission rate β within the SEITR model, where values greater than one suggest disease persistence and values less than one indicate elimination. An inverse and nonlinear relationship is evident between *R*_0_ and τ, with elevated reproduction levels present at low τ values, which quickly decline toward near-zero as τ increases, signifying improved epidemic management. A direct and roughly linear relationship is observed between *R*_0_ and β, where an increase in transmission intensity results in a proportional rise in the reproduction number. When β reaches a certain point, the epidemic threshold is crossed, and continuous transmission is expected. These findings underscore that both efficient transmission reduction and the improvement of treatment-related processes are essential for reducing *R*_0_ below the critical threshold and achieving disease control.

### 8.3. Dynamics of control strategies

**Fig 9:**
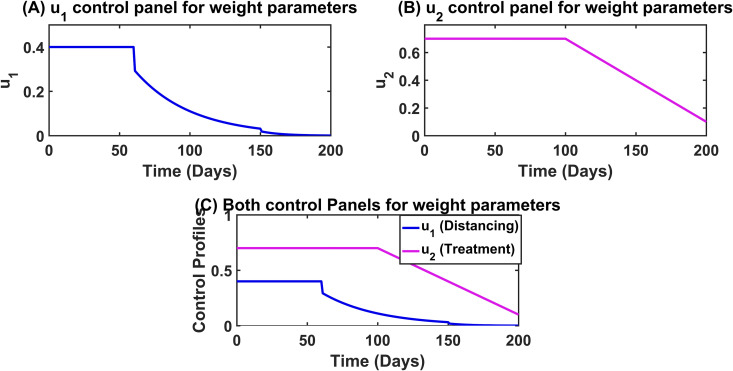
Different control panels (A), (B), and (C) obtained using different weight parameters.

The control panel profile is used to show how important intervention parameters change and work together in the proposed modeling structure. We examine the impact of control methods on system behaviour and their efficacy in infection management by demonstrating their functionality. The model uses the following controls, which are shown in the graphs below:

Fig 9 illustrate the most effective time-dependent methods for epidemic control. They demonstrate prevention (*u*_1_, labeled as Distancing) and treatment (*u*_2_, labeled as therapy) efforts over a 200-day period. The graphs demonstrate that an effective control technique necessitates both high levels of distance and therapy at the beginning. For the first 60 days, distance efforts (*u*_1_) should be held at 0.4 and then rapidly reduced. This means that preventative efforts should be prioritized from the start. In contrast, treatment efforts (*u*_2_) are maintained at a higher level of 0.7 for a longer period, typically 100 days, before decreasing progressively. This suggests a phased approach in which early and robust prevention measures are critical, followed by continuous therapeutic intervention, which is gradually reduced, to effectively control the epidemic, as modeled by an SEITR that is gradually reduced, compartmental system. The weight parameters (*a*_1_, *a*_2_, *a*_3_, *a*_4_, *a*_5_) determine the relative relevance of various objectives in the control issue. For example, a greater emphasis on therapy interventions may result. By comparing the control techniques in the three figures (Fig 9A, Fig 9B and Fig 9C (S1 Fig)), we can understand how changes in the weight parameters influence the best control options.

**Fig 10:**
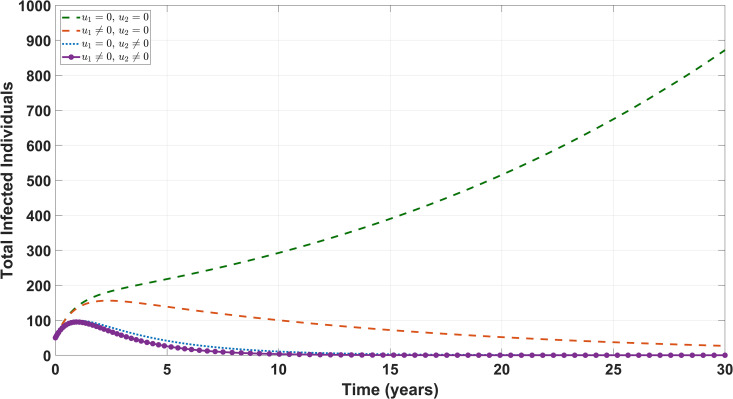
The density of total infected individuals analyzed with and without control measures.

Fig 10 (S1 Fig) illustrates the results of a mathematical model for tuberculosis (TB), which integrates various disease control measures over time. This model examines four scenarios for preventing the spread of TB using two types of interventions: distance control (*u*_1_) and treatment control (*u*_2_). The green dashed line ($u_{1}=0,u_{2}=0$) represents a scenario with no control measures implemented (i.e., neither distancing nor treatment). In this case, the number of infected individuals consistently increases over time, demonstrating the worst-case scenario in which the disease spreads uncontrollably. The red dashed line ($u_{1}\neq 0,u_{2}=0$) indicates a scenario in which only distance control is applied, without any treatment control. In this case, the number of infections decreases below the level shown by the green line, but not as significantly as in other scenarios. While distancing helps slow the spread of the disease, it cannot completely contain the outbreak.

The blue dotted line ($u_{1}=0,u_{2}\neq 0$) indicates that only treatment control is implemented, while no distancing control is applied. In this scenario, the number of infections initially increases; however, over time, treatment significantly reduces the infected population. This method proves to be more effective than relying solely on distancing measures. On the other hand, the purple line with circles ($u_{1}\neq 0,u_{2}\neq 0$) shows that both distancing and treatment controls are applied simultaneously. This combined approach results in the best outcomes, as infections are reduced rapidly and sustainably. This demonstrates that the most effective way to control tuberculosis (TB) is by combining both distancing and treatment strategies.

## 9. Cost-effectiveness evaluation

To enhance policy relevance, an Incremental Cost-Effectiveness Ratio (ICER) framework is introduced. The total intervention cost over the time horizon *T* is defined as


$$\begin{array}{c} C(u_{1},u_{2})=\int_{0}^{T}\left(\frac{a_{4}}{2}u_{1}^{2}+\frac{a_{5}}{2}u_{2}^{2}\right)\;dt. \end{array}$$

The effectiveness measure is defined as the total infections averted


$$\begin{array}{c} E=\int_{0}^{T}\left(I_{\text{no control}}-I_{\text{control}}\right)\;dt. \end{array}$$

The ICER is computed as


$$\begin{array}{c} \text{ICER}=\frac{C_{\text{strategy}}-C_{\text{baseline}}}{E_{\text{strategy}}-E_{\text{baseline}}}. \end{array}$$

According to the economic assessment in S3 Table, under the baseline scenario, no funds are allotted, hence no illnesses are avoided. Distancing alone results in a moderate decrease in TB cases, but the incremental cost per case is significantly higher than with treatment-based interventions. While the combination control approach yields the highest epidemiological benefit at a higher total cost, the treatment-only strategy is linked to the lowest cost per case avoided. From a Bangladesh perspective, the treatment-focused intervention is economically preferable if it costs less per TB case prevented, whereas the dual strategy may be justified when maximizing disease reduction is prioritized over cost minimization.

## 10. Conclusions

A numerical SEITR model is developed to investigate tuberculosis transmission and assess the effectiveness of distancing and treatment controls. The influence of key transmission and progression parameters on the basic reproduction number (*R*_0_) is examined to identify factors that sustain disease spread, and model stability has been analytically verified. Furthermore, optimal control theory is applied to evaluate strategies for reducing infection levels and to interpret the epidemiological implications of the results.

- The SEITR model is analyzed analytically and numerically. And it is established that the disease-free equilibrium is both locally and globally asymptotically stable whenever *R*_0_ < 1, while a unique endemic equilibrium is present and globally stable when *R*_0_ > 1.
- Evidence shows that reducing the effective contact rate (β) through distancing measures substantially inhibits transmission of new infections.
- Better treatment has been shown to speed up recovery and lower the death rate among people who are infected.
- The simultaneous implementation of distancing (*u*_1_) and treatment (*u*_2_) strategies has proven more effective than methods relying on a single intervention.
- It has been recognized that the timely and ongoing implementation of control measures is crucial for maintaining disease stability.
- The model’s alignment with actual data has been validated, reinforcing its importance for public health strategy development.
- The cost-effectiveness analysis showed that dual control has a higher total implementation cost, but it has the lowest additional cost per infection avoided. Consequently, the integrated control technique is acknowledged as economically superior under reasonable assumptions.

It is clear that the best way to control tuberculosis is to use both behavioural and medicinal treatments together. The models’ outcomes emphasize the significance of ongoing public awareness, access to health care, and adherence to treatment. Mathematical modeling has demonstrated efficacy as a decision-support instrument for optimizing intervention design and resource allocation. Optimal control simulations demonstrated that the simultaneous implementation of distance and treatment strategies yielded the greatest and most significant reduction in infection prevalence over time. Validation of the model using tuberculosis data from 2010 to 2025 demonstrated robust predictive consistency, and projection analysis indicated that the continued application of dual-control methods might substantially reduce the TB burden by 2030.

The model does not consider treatment failure or recurrence because this study assumes treatment is entirely effective. Therefore, some characteristics of true TB progression and control may be missed. The current SEITR optimum control model requires homogeneous mixing, consistent intervention adherence, and deterministic cost-effectiveness evaluation. It also lacks population heterogeneity, random variability, and uncertainty treatment, which may limit its real-world usefulness. For realism and policy relevance, treatment failure and relapse, varied contact patterns, stochastic dynamics, and larger economic evaluations will be integrated in future research.

## Supporting information

S1 Data Annual TB data used in this study. (XLSX)

S1 Fig Model flow diagram and supporting figure. (DOCX)

S1 Table Indices of sensitivity of *R*_0_ to some model parameters. (PDF)

S2 Table Global sensitivity results based on SRC and PRCC (N = 1000 samples). (PDF)

S3 Table Cost-effectiveness analysis of TB control strategies. (PDF)

S1 Appendix Overview of model parameters. (PDF)

S1 Text Annual Tuberculosis totals from 2010 to 2025. (PDF)
