# Supplementary material for: Dynamical behavior analysis of 2-control strategies on tuberculosis model
Source: PLOS Glob Public Health. 2026 Jun 8;6(6):e0005875. doi: 10.1371/journal.pgph.0005875 (PMC13245803; doi:10.1371/journal.pgph.0005875)
Supplement: S1 Fig — (DOCX) [file pgph.0005875.s002.docx]

**S1 Fig: Model flow diagram and supporting figure.**


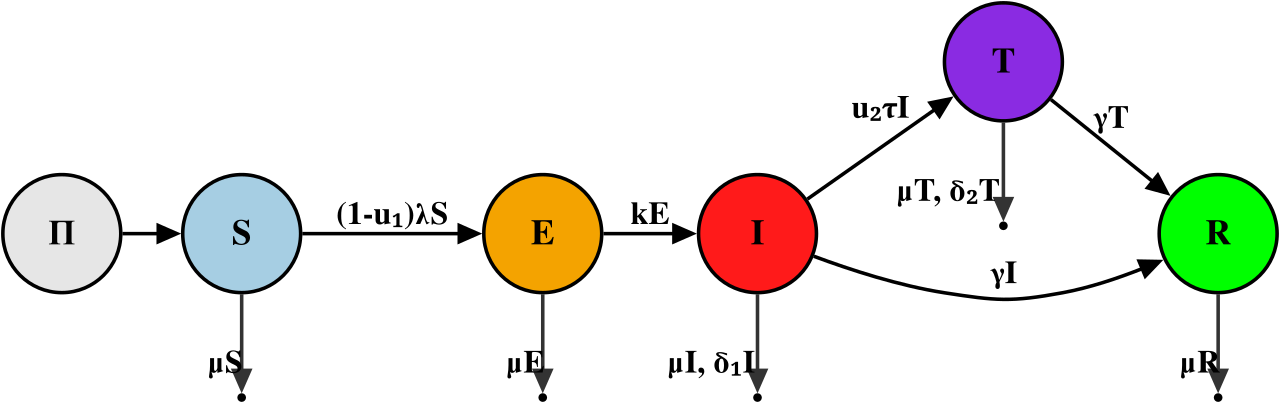


**Fig 1. Diagram of TB transmission with control strategies.**


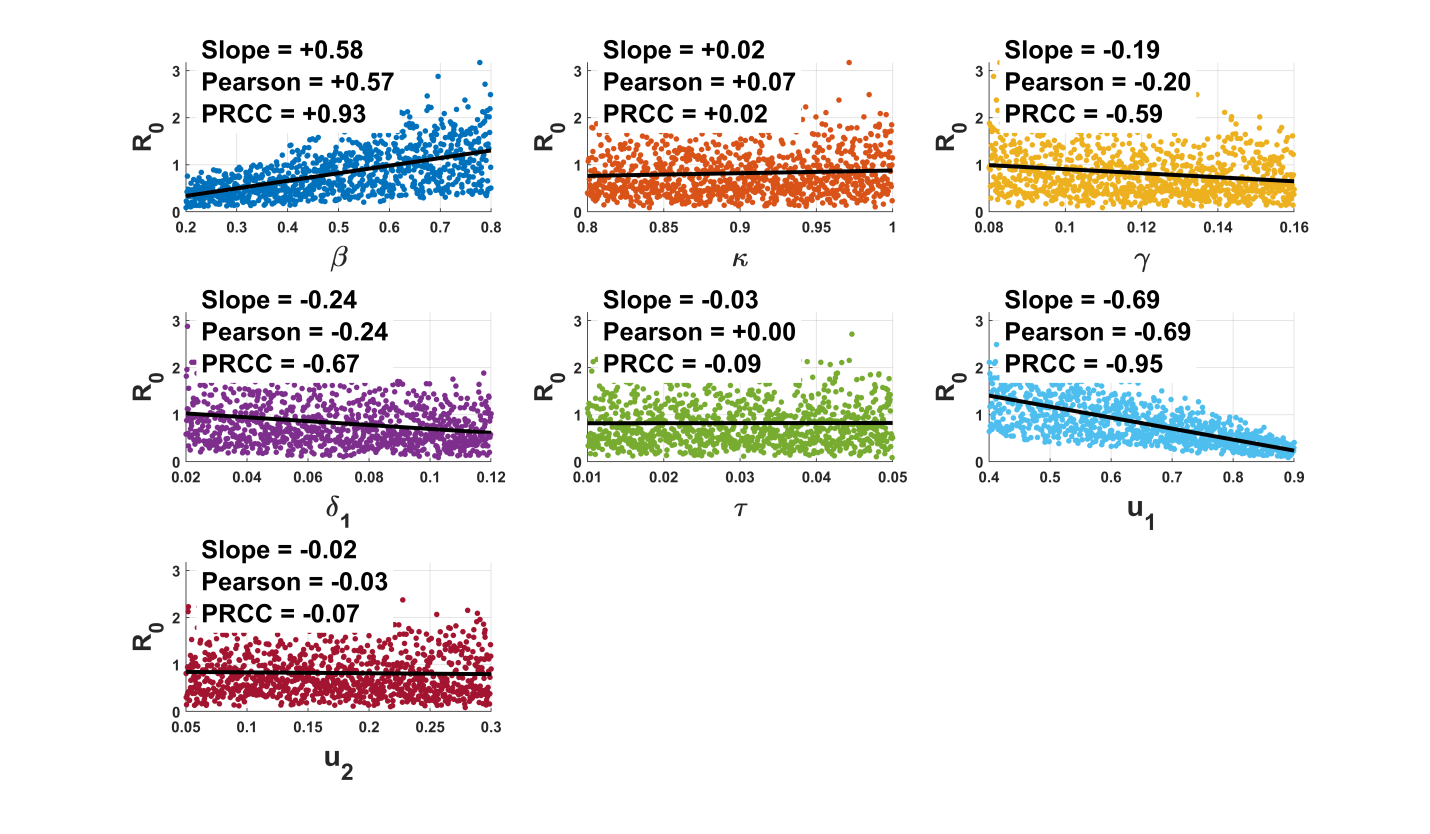


**Fig 2. Regression, Pearson and PRCC analysis of model parameters by scatter diagram.**


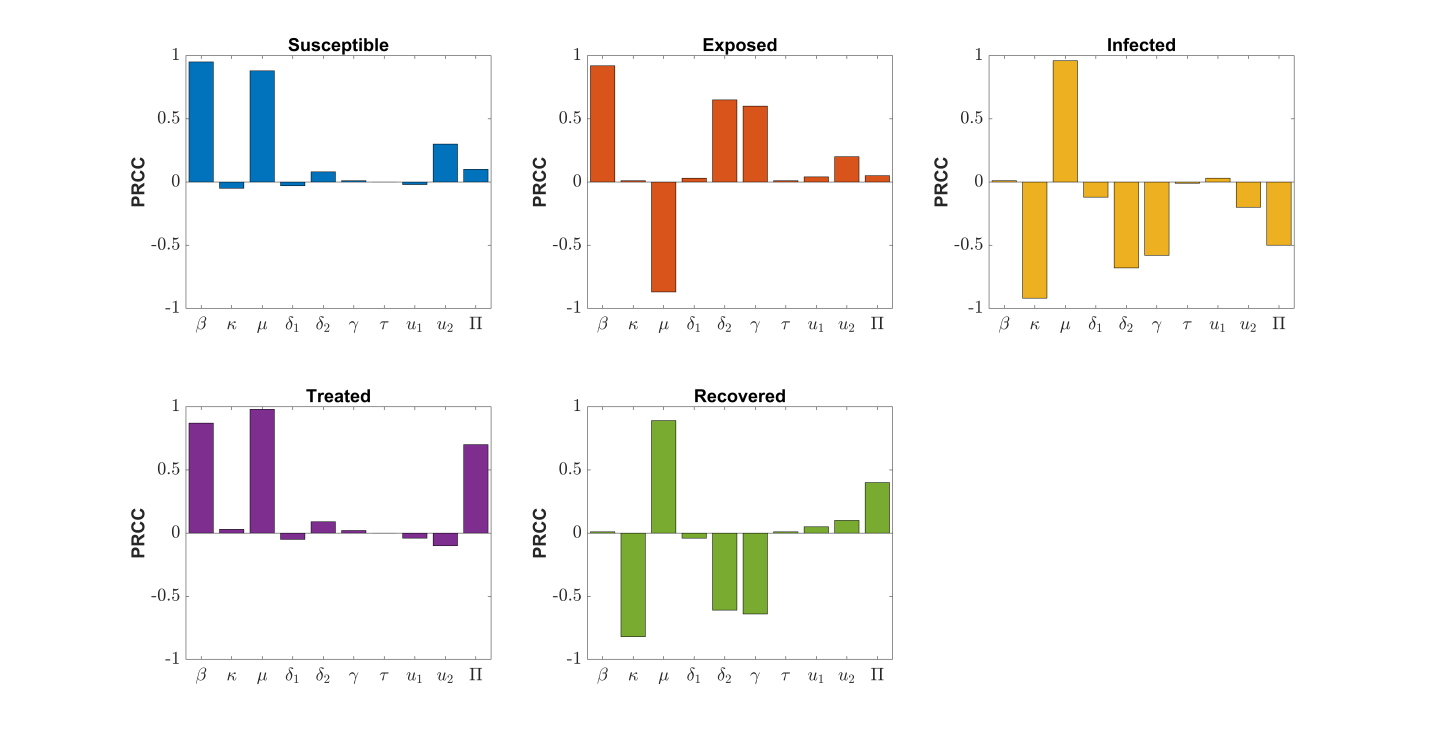
**Fig 3. PRCC indices of all parameters for each compartments of the model.**


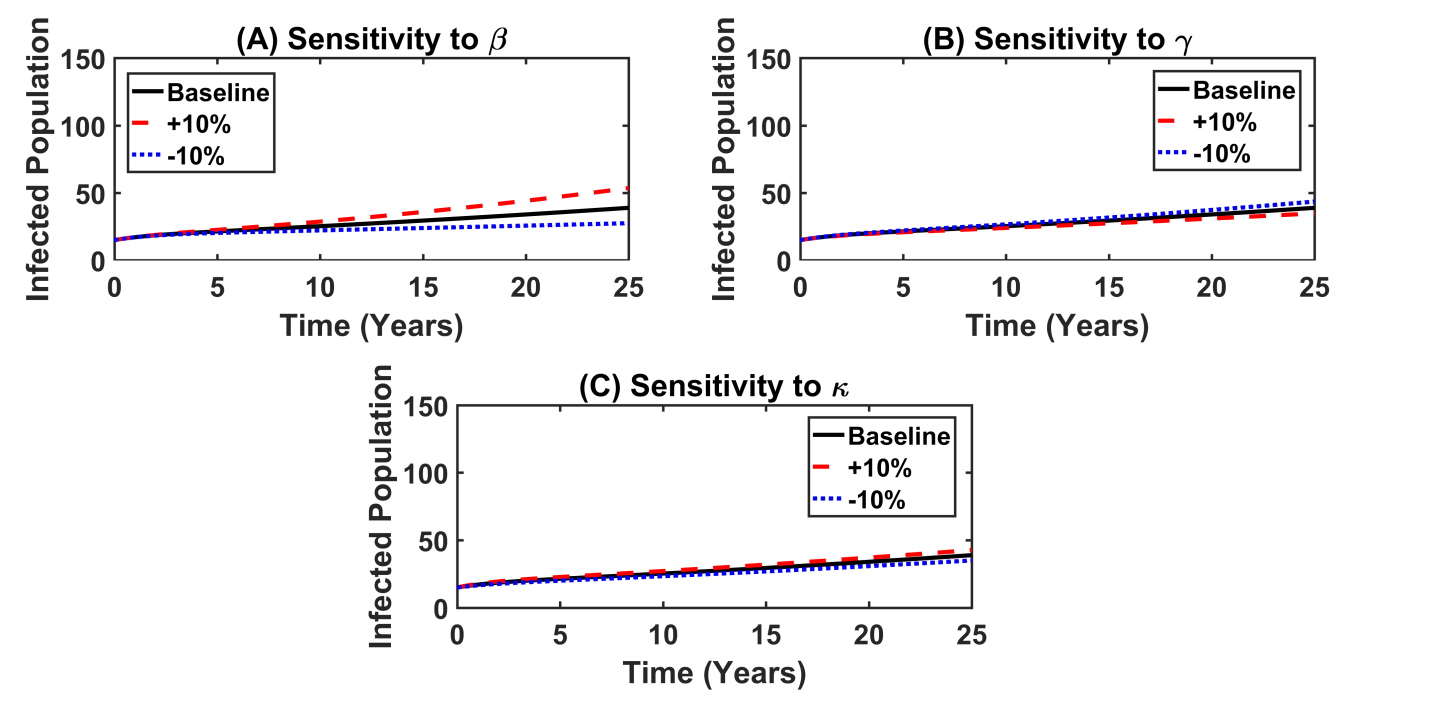


**Fig 4. Sensitivity analysis impact on infectiousness for β, γ, and κ.**


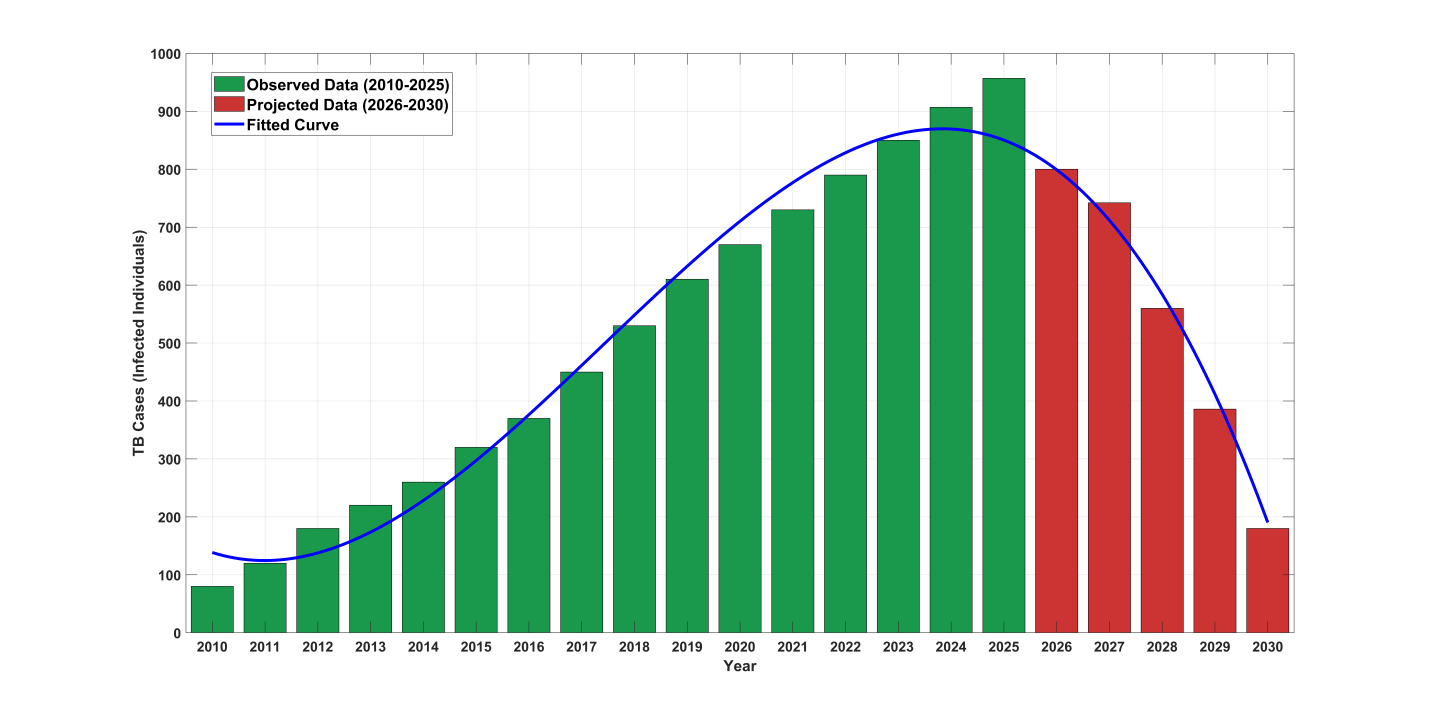
**Fig 5.** **Curve fitting of infected cases with model prediction with projection up to 2030.**


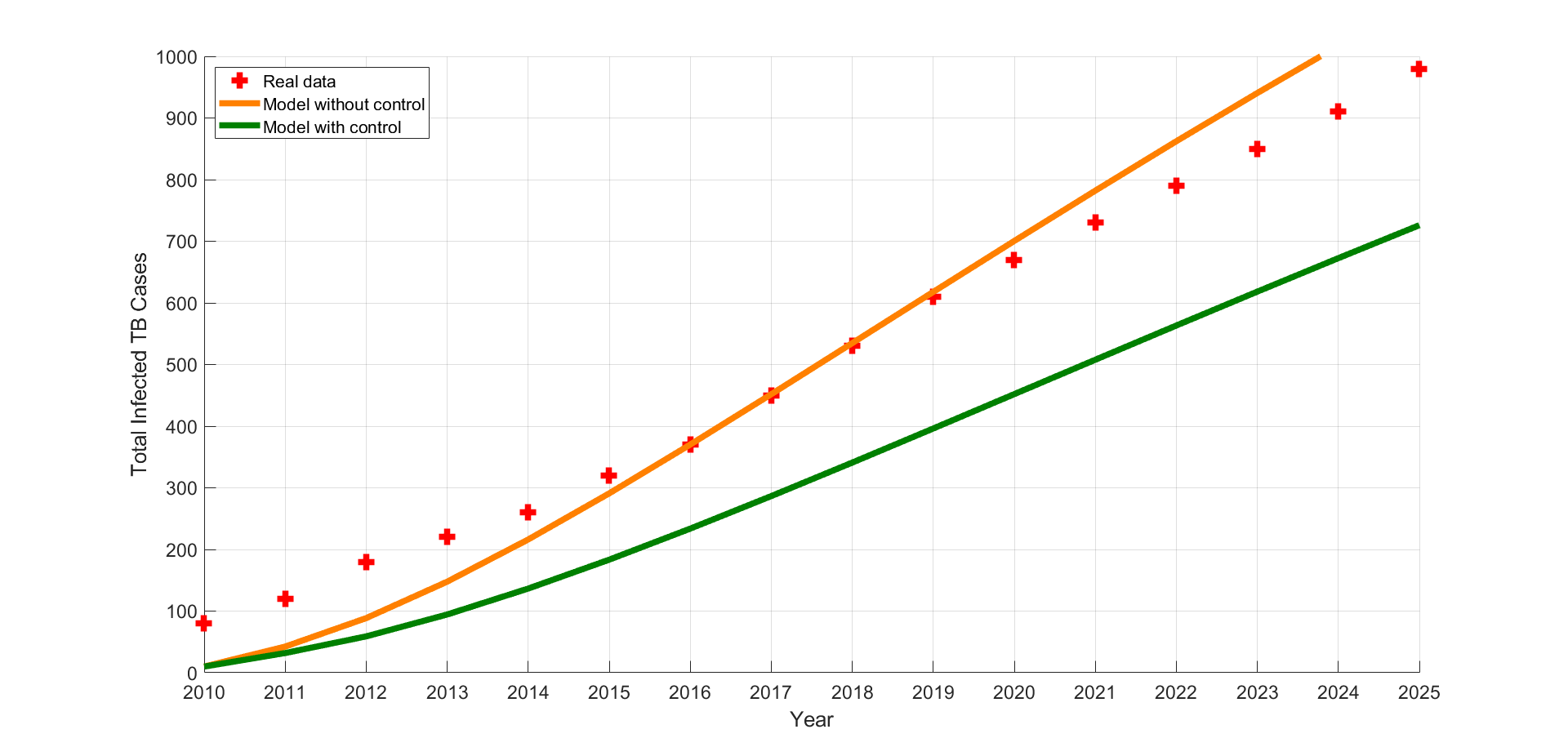


**Fig 6. Model Fit to Real Data (2010-2025) of total TB cases assessed with and without control measures.**


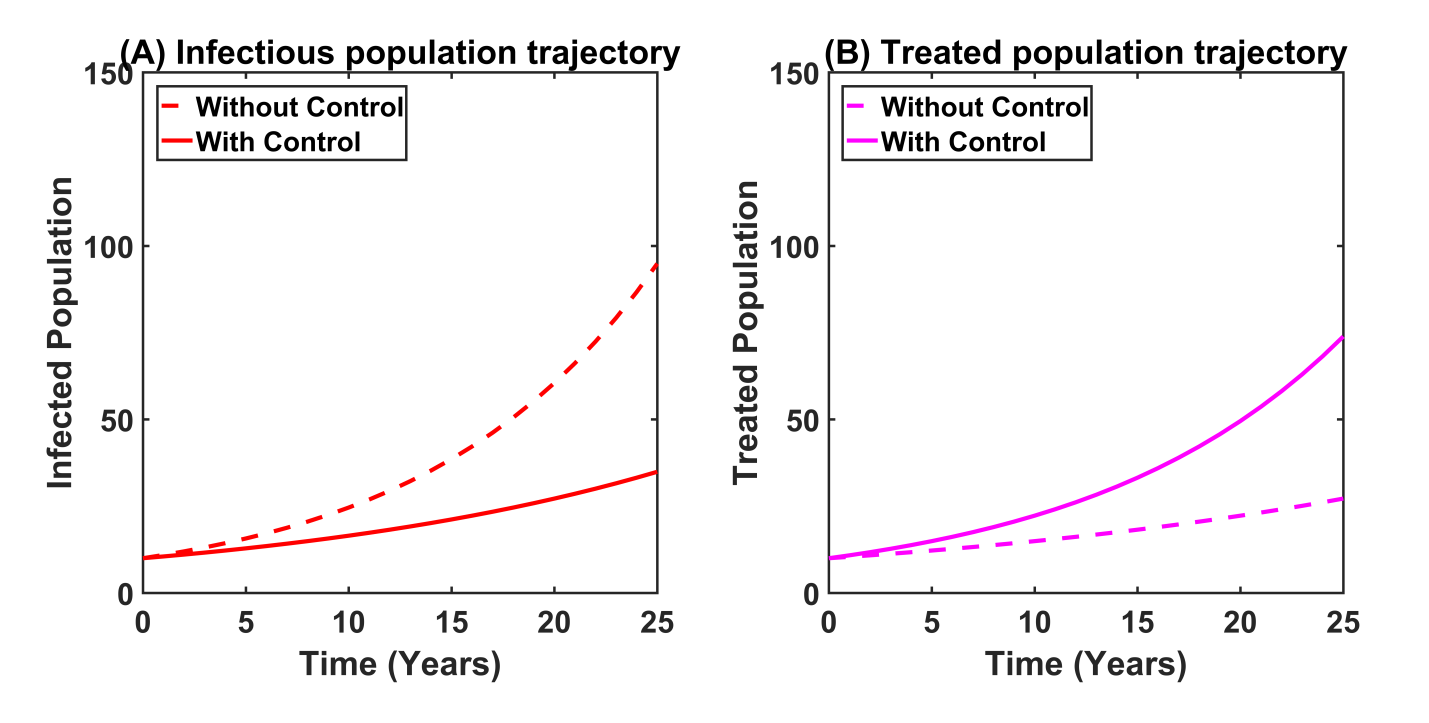
**Fig 7. Comparison of infectious and treated population trajectories under controls.**


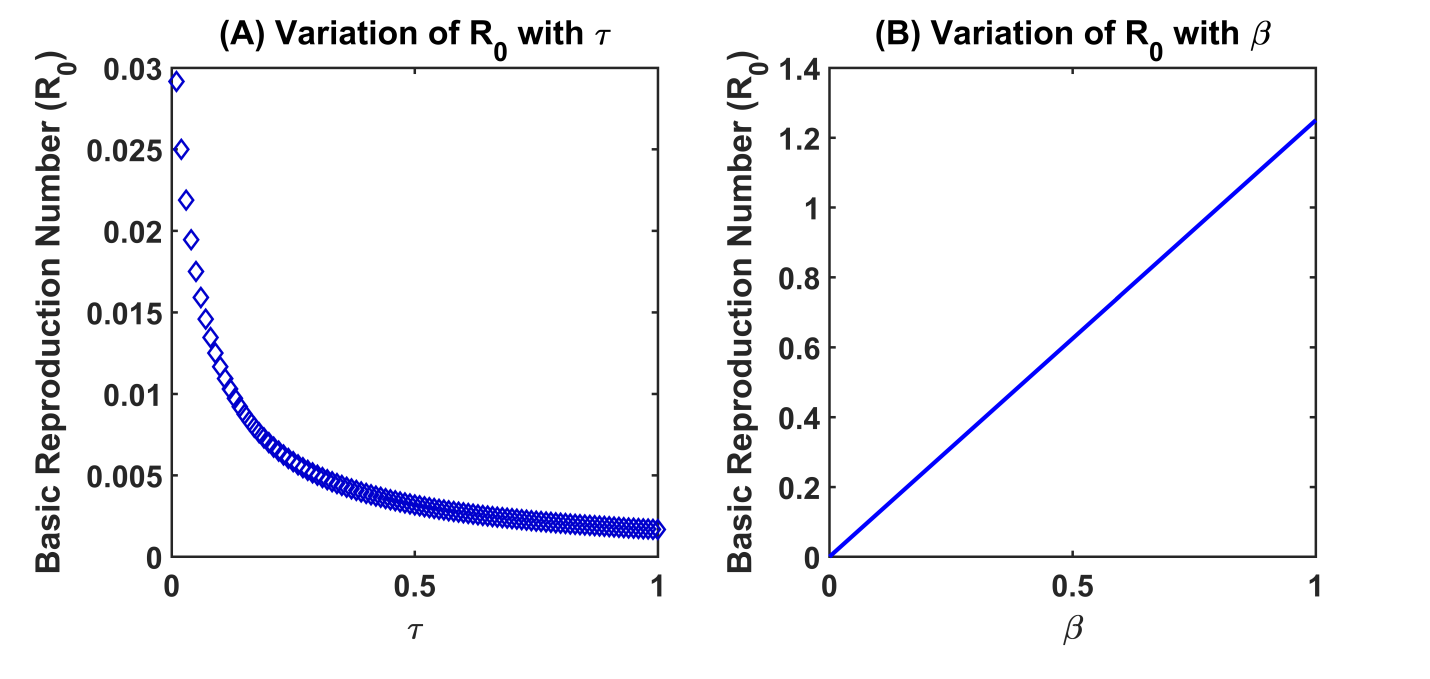
**Fig 8.** **Sensitivity of the basic reproduction number (R_0_) with respect to parameters τ and β.**


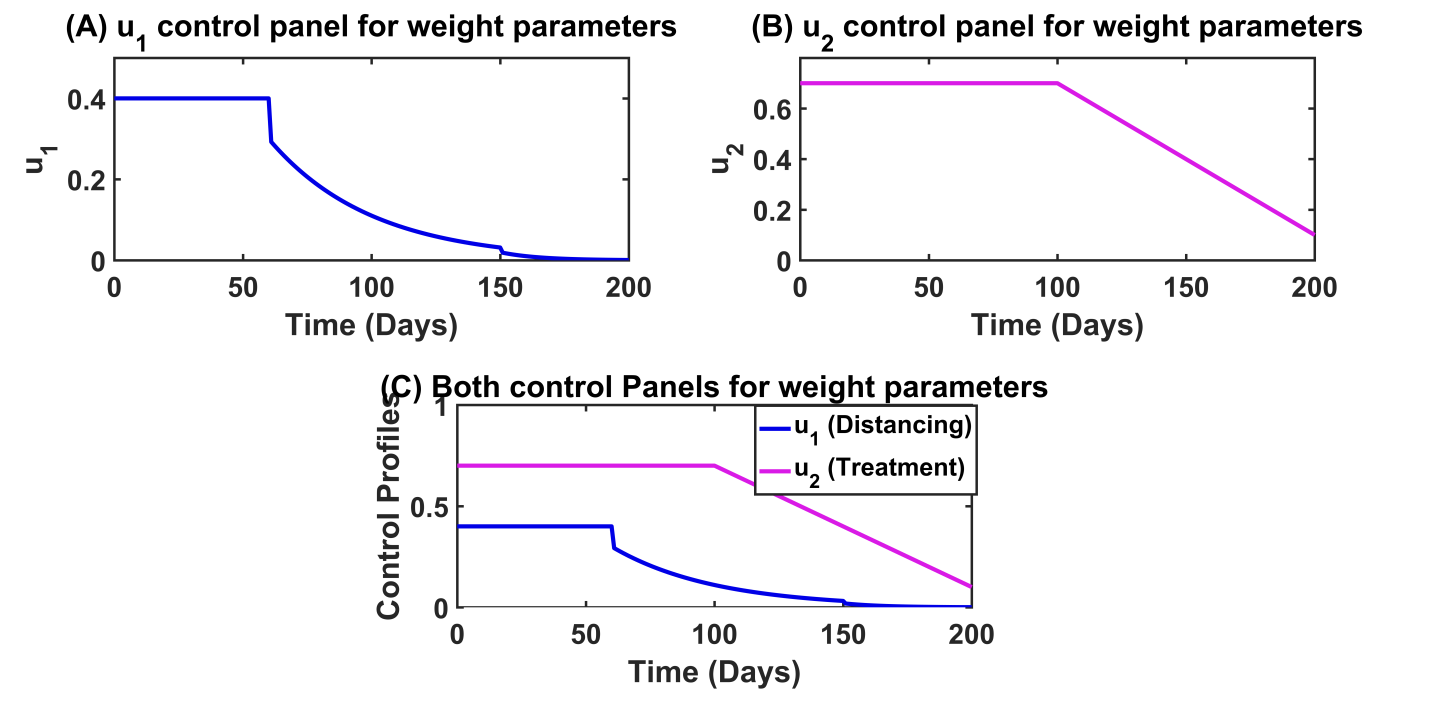
**Fig 9. Different control panels (A), (B), and (C ) obtained using different weight parameters.**


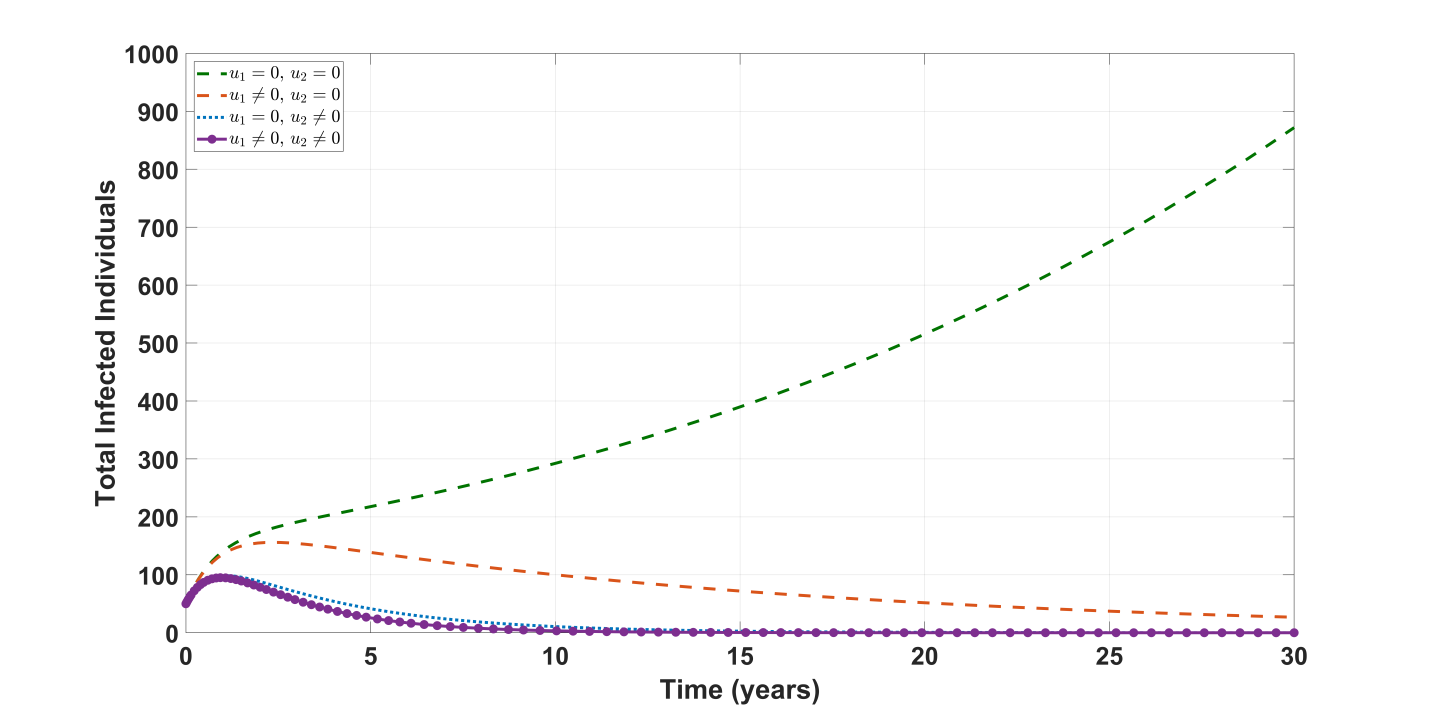
**Fig10. The density of total infected individuals analyzed with and without control measures.**
