# Supplementary material for: Dynamical behavior analysis of 2-control strategies on tuberculosis model
Source: PLOS Glob Public Health. 2026 Jun 8;6(6):e0005875. doi: 10.1371/journal.pgph.0005875 (PMC13245803; doi:10.1371/journal.pgph.0005875)
Supplement: S1 Table — (PDF) [file pgph.0005875.s003.pdf]

S1 Table. Indices of sensitivity of R_0 to some model parameters

Parameters	Indices of sensitivity
β	0.8714
κ	0.0260
γ	-0.6195
τ	-0.0728
δ_1	-0.6457
u_1	-0.9159
u_2	-0.2765
