# Supplementary material for: Dynamical behavior analysis of 2-control strategies on tuberculosis model
Source: PLOS Glob Public Health. 2026 Jun 8;6(6):e0005875. doi: 10.1371/journal.pgph.0005875 (PMC13245803; doi:10.1371/journal.pgph.0005875)
Supplement: S2 Table — (PDF) [file pgph.0005875.s004.pdf]

S2 Table. Global sensitivity results based on SRC and PRCC (N=1000 samples)

Parameter	Range	PRCC	Slope	95% CI	p-value	Effect on R0
u_1	0.4–0.9	-0.95354	-0.69	(-0.95885 , -0.94756)	$<10^{-300}$	Very strong negative
β	0.2–0.8	0.93028	+0.58	(0.92143 , 0.93817)	$<10^{-300}$	Very strong positive
δ_1	0.02–0.12	-0.66736	-0.24	(-0.70038 , -0.63149)	$<10^{-120}$	Strong negative
γ	0.2–0.4	-0.59017	-0.19	(-0.62915 , -0.54823)	$<10^{-90}$	Strong negative
τ	0.5–0.8	-0.08754	-0.03	(-0.14872 , -0.02568)	0.0056	Weak negative
u_2	0.05–0.3	-0.07258	-0.02	(-0.13397 , -0.01064)	0.022	Weak negative
κ	0.8–1.00	0.02396	+0.02	(-0.03809 , 0.08583)	0.44	Not significant
