# Supplementary material for: Dynamical behavior analysis of 2-control strategies on tuberculosis model
Source: PLOS Glob Public Health. 2026 Jun 8;6(6):e0005875. doi: 10.1371/journal.pgph.0005875 (PMC13245803; doi:10.1371/journal.pgph.0005875)
Supplement: S3 Table — (PDF) [file pgph.0005875.s005.pdf]

S3 Table. Cost-effectiveness analysis of TB control strategies

Strategy	Total Cost (BDT)	Cases Averted	ICER (BDT per case)	Interpretation
No control	0	0	---	Baseline reference
Distancing only (u_1)	9.70×10^5	1387.5	699.37	Moderately cost-effective but less efficient
Treatment only (u_2)	4.84×10^5	1942.5	248.90	Most cost-effective strategy
Dual control (u_1,u_2)	1.45×10^6	2913.8	498.97	Highly effective but higher cost per case
