# Supplementary material for: Dynamical behavior analysis of 2-control strategies on tuberculosis model
Source: PLOS Glob Public Health. 2026 Jun 8;6(6):e0005875. doi: 10.1371/journal.pgph.0005875 (PMC13245803; doi:10.1371/journal.pgph.0005875)
Supplement: S1 Appendix — (PDF) [file pgph.0005875.s006.pdf]

S1 Appendix: Overview of model parameters

Parameter	Biological Meaning	Value and Unit	Source
μ	Natural death rate	0.02 $year^{-1}$	Nayeem & Sultana (2019)
δ_1	Disease-induced death rate (acute infected)	0.1 $year^{-1}$	Assumed
δ_2	Disease-induced death rate (treatment class)	0.1 $year^{-1}$	Assumed
κ	Progression rate of exposed class	0.85	Nayeem & Sultana (2019)
τ	Treatment rate	0.8	Nayeem & Sultana (2019)
γ	Recovery rate	0.23 $year^{-1}$	Nayeem & Sultana (2019)
Π	Constant recruitment rate	2000 individuals $year^{-1}$	Assumed
β	Transmission rate	0.45 $year^{-1}$	Nayeem & Sultana (2019)
