# Supplementary material for: Dynamical behavior analysis of 2-control strategies on tuberculosis model
Source: PLOS Glob Public Health. 2026 Jun 8;6(6):e0005875. doi: 10.1371/journal.pgph.0005875 (PMC13245803; doi:10.1371/journal.pgph.0005875)
Supplement: S1 Text — (PDF) [file pgph.0005875.s007.pdf]

# Supporting Information_1

**S1 Text. Annual Tuberculosis totals from 2010 to 2025.**

| Year | Real data annual total | Model without control (approx.) | Model with control (approx.) |
|---|---|---|---|
| 2010 | 81 | 10 | 10 |
| 2011 | 121 | 43 | 32 |
| 2012 | 182 | 89 | 60 |
| 2013 | 224 | 150 | 96 |
| 2014 | 265 | 220 | 139 |
| 2015 | 325 | 295 | 186 |
| 2016 | 376 | 376 | 238 |
| 2017 | 456 | 459 | 292 |
| 2018 | 538 | 543 | 347 |
| 2019 | 618 | 628 | 403 |
| 2020 | 681 | 713 | 460 |
| 2021 | 744 | 796 | 516 |
| 2022 | 804 | 877 | 573 |
| 2023 | 865 | 957 | 629 |
| 2024 | 927 | 1000 | 684 |
| 2025 | 997 | | 737 |

https://www.who.int/teams/global-tuberculosis-programme/data and https://data.worldbank.org/indicator/SH.TBS.INCD.
