# Supplementary material for: Dynamical behavior analysis of 2-control strategies on tuberculosis model
Source: PLOS Glob Public Health. 2026 Jun 8;6(6):e0005875. doi: 10.1371/journal.pgph.0005875 (PMC13245803; doi:10.1371/journal.pgph.0005875)
Supplement: Attachment — Submitted filename: Review Comments for PLOS HEALTH REVIEW.docx [file pgph.0005875.s008.docx]

**Dear Editor**

**PLOS Global Public Health (PGPH-D-26-00093)**

**Reviewer’s Comments**

The authors studied ‘**Dynamical Behavior Analysis of 2-control Strategies on Tuberculosis Model**. The authors must be commended for carrying out this research. As the paper is, technically correct formulation of the SEITR system, Standard application of Pontryagin’s Maximum Principle, Inclusion of sensitivity analysis and data fitting and clear policy motivation (treatment and distancing synergy). However, authors need to address the following suggestions to improve the manuscript:

**Comment 1: Abstract**

Please consider/incorporate the followings points in the abstract

i. aim/ objectives

ii. Methods applied

iii. Results/ Findings

iv. Recommendations to policymakers

**Comment 2: Introduction**

Please consider/incorporate the following points in the introduction.

i. Significance of the research

ii. Please clearly indicate in a summarized form your observations about the related literature reviewed as compared to your research.

**Comment 3: Research gap and main contribution**

At the ending section of the introduction:

i. Authors should clearly state the research gap in the manuscript.

ii. SEIR/SEITR TB models with: treatment control, distancing or awareness control, PMP-based optimal control, sensitivity analysis have been extensively studied (including several papers already cited). Authors should compare their research with the existing literature and state what exactly is new in their research (main contribution) compared to existing studies.

iii. The claimed novelty which comprises “composite analytical methodology integrating control-oriented stability decomposition with normalized forward sensitivity indexing” is not clearly demonstrated mathematically. Authors should check that.

**Comment 4: Stability analysis**

The stability analysis must be improved. Authors analyzed only DFE stability. Authors should consider Endemic equilibrium (EE) existence, Local or global stability of EE and Backward bifurcation analysis. Since TB models, normally exhibit backward bifurcation and highly relevant.

**Comment 5: Sensitivity analysis**

Authors conducted so many sensitivity plots, with little insight. Parameter ranges are not clearly justified and some interpretations are inconsistent (e.g., γ showing weak PRCC but strong analytical sensitivity). Authors should correct that.

**Comment 6: Grammatical Errors**

There are some grammatical errors in the manuscript. For instance

1. “Pontryagain’s” → **Pontryagin’s**

2. Repetitive phrases (“It is noted that…”, “It is observed that…”) and

3. Overly long descriptive captions instead of analytical discussion

**Comment 7: Inconsistencies in Notations**

1. The authors used \lambda for both force of infection and adjoint derivative,
2. Parameters appear in equations but are not always defined immediately
3. Table 1 includes parameter \eta which does not appear in the model

Authors should correct that.

**Comment 9: In Text-Reference**

A lot of in-text references are missing in the manuscript. Authors should correct that.

**Comment 10: Conclusion**

State the limitations and future work in this section.

**Comment 11: Cost-effectiveness analysis**

Cost-effectiveness analysis can be added to the control model to improve the manuscript.

**Remarks**: Minor Revision Required

Thank you
