# Supplementary material for: Dynamical behavior analysis of 2-control strategies on tuberculosis model
Source: PLOS Glob Public Health. 2026 Jun 8;6(6):e0005875. doi: 10.1371/journal.pgph.0005875 (PMC13245803; doi:10.1371/journal.pgph.0005875)
Supplement: Attachment — Submitted filename: Response to Reviewers.docx [file pgph.0005875.s010.docx]

**Title:** Dynamical Behavior Analysis of 2-control Strategies on Tuberculosis Model
**Paper ID: PGPH-D-26-00093**
PLOS Global Public Health.

**Dear Editor and Reviewers**

We sincerely thank the Editor and all Reviewers for their careful evaluation of our manuscript and for their insightful and constructive comments. We have thoroughly revised the manuscript in response to all suggestions. We believe these revisions have significantly strengthened the clarity, transparency, rigor, and overall quality of our work.

Below, we respond to each comment individually.

**Response to the Editor**

| **Q1.** | Please note that PLOS Global Public Health has specific guidelines on code sharing for submissions in which author-generated code underpins the findings in the manuscript. In these cases, all author-generated code must be made available without restrictions upon publication of the work. Please review our guidelines at <https://journals.plos.org/globalpublichealth/s/materials-and-software-sharing#loc-sharing-code> and ensure that your code is shared in a way that follows best practice and facilitates reproducibility and reuse. |
| --- | --- |
| **Ans.** | We thank the Editor for emphasizing the PLOS code-sharing policy and fully support the importance of transparency and reproducibility in computational research. All relevant data and source code in the revised manuscript are provided in the Supporting Information as a **Data and Code.zip file.** |
| **Q2.** | In the ethics statement in the Methods, you have specified that verbal consent was obtained. Please provide additional details regarding how this consent was documented and witnessed, and state whether this was approved by the IRB. |
| **Ans.** | Thank you very much to the Editor for the comment. The study used publicly available aggregated tuberculosis data from the WHO Global Tuberculosis Programme database (https://www.who.int/teams/global-tuberculosis-programme/data), and no direct human participation or individual consent was required; additionally, ethical approval was not required according to institutional guidelines. |
| **Q3.** | Please provide a detailed online Financial Disclosure statement. This is published with the article. It must therefore be completed in full sentences and contain the exact wording you wish to be published.  a) State the initials, alongside each funding source, of each author to receive each grant. For example: “This work was supported by the National Institutes of Health (####### to AM; ###### to CJ) and the National Science Foundation (###### to AM).”  b) State what role the funders took in the study. If the funders had no role in your study, please state: “The funders had no role in study design, data collection and analysis, decision to publish, or preparation of the manuscript.”  For more information, please go to our submission guidelines: <https://journals.plos.org/globalpublichealth/s/submission-guidelines#loc-financial-disclosure-statement> |
| **Ans.** | We have updated the manuscript to include a complete Financial Disclosure statement as required by PLOS in the **Funding Section**. |
| **Q4.** | Please ensure that the funders and grant numbers match between the Financial Disclosure field and the Funding Information tab in your submission form. Note that the funders must be provided in the same order in both places as well. |
| **Ans.** | Thank you to the editor for raising this point. We have thoroughly verified the Funding section in the revised manuscript. |
| **Q5.** | Please update your online Competing Interests statement. If you have no competing interests to declare, please state: “The authors have declared that no competing interests exist.” |
| **Ans.** | The Competing Interests statement has been updated to:  “The authors have declared that no competing interests exist.” |
| **Q6.** | Thank you for uploading your study's underlying data set. Unfortunately, the repository you have noted in your Data Availability statement does not qualify as an acceptable data repository according to PLOS's standards.  At this time, please upload the minimal data set necessary to replicate your study's findings to a stable, public repository (such as figshare or Dryad) and provide us with the relevant URLs, DOIs, or accession numbers that may be used to access these data. For a list of recommended repositories and additional information on PLOS standards for data deposition, please see <https://journals.plos.org/globalpublichealth/s/recommended-repositories>. |
| **Ans.** | We appreciate the clarification regarding acceptable repositories.  The minimal dataset necessary to reproduce our findings has now been uploaded to the Supporting Information. |
| **Q7.** | We note that you may not have used the PLOS LaTeX template. This is not required, but please be sure that you are familiar with our LaTeX guidelines to help reduce the chance of delay due to typesetting errors. Our guidelines can be found here: <https://journals.plos.org/digitalhealth/s/latex> |
| **Ans.** | We have thoroughly reviewed the LaTeX guidelines to ensure that the formatting, citations, equations, and structure comply with the journal's standards, thereby avoiding typesetting delays. |
| **Q8.** | Please ensure that you have provided a single, cohesive.tex file for your LaTeX submission. As stated in the PLOS template, your references should be included in your .tex file (not submitted separately as .bib or .bbl). Please also ensure that you are making any formatting changes to both your .tex file and the PDF of your manuscript. If you have any questions, please contact [Latex@plos.org](mailto:Latex@plos.org). You can find our LaTeX guidelines here: <https://journals.plos.org/digitalhealth/s/latex> |
| **Ans.** | We confirm that a single cohesive .tex file has been provided, with references embedded within the file and all formatting changes fully synchronized with the submitted PDF version. |
| **Q9.** | Please provide separate main figure files in .tif or .eps format only and ensure that all files are under our size limit of 10MB. You may leave the embedded figures in the manuscript.  For more information about how to convert your figure files please see our guidelines: <https://journals.plos.org/globalpublichealth/s/figures> |
| **Ans.** | All main figures have now been uploaded separately in .eps format. |
| **Q10.** | We notice that your supplementary material (Appendix) is included in the manuscript file. Please remove them and upload them with the file type 'Supporting Information'. Please ensure that each Supporting Information file has a legend listed in the manuscript before or after the references list. |
| **Ans.** | The supplementary material has been removed from the main manuscript and uploaded separately under “Supporting Information.” |
| **Q11.** | If the reviewer comments include a recommendation to cite specific previously published works, please review and evaluate these publications to determine whether they are relevant and should be cited. There is no requirement to cite these works unless the editor has indicated otherwise. |
| **Ans.** | We thoroughly reviewed all suggested citations. Relevant and scientifically appropriate references have been included. Citations are not added when they are not directly relevant, in accordance with journal policy. |

**Response to the Reviewer #1**

| **Q1.** | The modeling approach is fundamentally sound and relevant to TB control (a major public health issue), but the manuscript needs to address transparency (data sources, parameter justification) and clarify some methodological assumptions (e.g. definition of Reproduction number) before it can be accepted. |
| --- | --- |
| **Ans.** | We appreciate the reviewer for acknowledging the importance of our modeling framework. We have revised the manuscript by clearly defining the basic reproduction number $R_{0}$ (**Section 3.2**), parameter justification with relevant literature (**Appendix**), clarifying data sources (**Supporting Information**), and improving overall methodological transparency (**Subsection 8.1**). |
| **Q2.** | The sensitivity analysis methods (Pearson, PRCC) are suitable but need more rigor in reporting (sample details, uncertainty). The data-fitting procedure requires complete description (data source, fitting algorithm, goodness-of-fit). The authors should include more quantitative statistical assessment of their model fit and uncertainty in parameter effects. |
| **Ans.** | Thank you very much to the reviewer for the helpful suggestion. According to the suggestion the manuscript is revised. (Subsection 6.2 (Table 2), Section 7). |
| **Q3.** | The authors should provide a Data Availability Statement with actual access information (URL, DOI, or supplementary files) for the epidemiological data and any other material used. At the end of the file, an editorial link points to the Bangladesh NTP Annual Report 2022, which presumably provided TB case counts, but this is not an open dataset citation. Without accessible data, readers cannot reproduce or verify the results. |
| **Ans.** | We have now provided full access information (DOI and URL) to the deposited dataset in the Supporting Information file. |
| **Q4.** | The writing requires major language editing. Grammatical issues and awkward phrasing occur repeatedly. Several citations are incomplete (marked as “[?]”). These issues hinder readability. The manuscript would benefit from careful editing by a fluent English speaker. With editing, the intellectual content is conveyable, but in its current state the language detracts from clarity. |
| **Ans.** | Thank you very much for your question. Now the manuscript has been thoroughly checked by English languish Professor. All grammatical errors, awkward phrasing, and incomplete citations are corrected in the revised manuscript. An English Language clarity certificate is uploaded in the Data and Code.zip file as a Supporting Information document. |

**Response to the Reviewer # 2**

| **Q1.** | Language and Presentation The manuscript contains numerous grammatical errors, awkward phrasing, and unclear sentences that affect readability. Several sections require substantial English language editing. Professional proofreading is strongly recommended to improve clarity and coherence. |
| --- | --- |
| **Ans.** | Thank you very much for your question. Now the manuscript has been thoroughly checked by English languish Professor. All grammatical errors, awkward phrasing, and incomplete citations are corrected in the revised manuscript. An English Language clarity certificate is uploaded in the Data and Code.zip file as a Supporting Information document. |
| **Q2.** | Incomplete and Missing Citations Many references in the text are marked with “[?]”, indicating missing citations. These must be replaced with appropriate and verifiable references. Failure to do so weakens the scientific credibility of the manuscript. |
| **Ans.** | All “[?]” placeholders have been replaced with verified and properly formatted references. |
| **Q3.** | Reference Quality and Formatting Several references appear incomplete, inconsistently formatted, or potentially inaccurate. All references should be verified and formatted according to journal guidelines. The authors should ensure that all cited sources are reliable and accessible. |
| **Ans.** | Thank you for your comment All references have been verified, completed, and reformatted according to PLOS guidelines. |
| **Q4.** | Mathematical Notation and Consistency Some notations are used ambiguously, particularly the symbol λ, which represents both the force of infection and adjoint variables. This may confuse readers. The notation should be revised for clarity. In addition, some derivations in the optimal control section are not sufficiently detailed and require clearer explanation. |
| **Ans.** | We have distinguished the force of infection from the adjoint variables (by using different notation) and expanded the derivations in the optimal control section.(Subsection 5.2) |
| **Q5.** | Data Availability and Reproducibility, The Data Availability Statement is insufficient. PLOS requires full access to underlying data and, where applicable, code. The authors should provide the dataset in an accessible repository and include links. Details on parameter estimation and model fitting procedures should also be expanded. |
| **Ans.** | We have now provided full access information (DOI and URL) to the deposited dataset in the Supporting Information file. And, the model parameters are estimated using **nonlinear polynomial regression with a least-squares optimization method with confidence interval.** (Subsection 6.2) |
| **Q6.** | Model Validation while curve fitting is presented, quantitative goodness-of-fit measures (e.g., RMSE, R², confidence intervals) are not provided. Including these metrics would strengthen the validation of the model. |
| **Ans.** | The goodness-of-fit of the model is evaluated using the **coefficient of determination (R² > 0.97),** indicating a strong agreement between the observed data and the model predictions. (Section 7) |
| **Q7.** | Overstatement of Novelty, The novelty section contains strong claims that are not fully supported by the analysis. The authors should moderate these statements and clearly specify how their approach differs from and extends existing studies. |
| **Ans.** | According to the reviewer’s comment, we effectively moderated claims of novelty and clearly defined our specific contributions without exaggeration in the last part of the introduction of the revised manuscript. [Section 1 at the end of page 2] |
| **Q8.** | Figures and Visualization, Some figures lack sufficient resolution, clear legends, and readable labels. All figures should be revised to meet publication standards and to improve interpretability. |
| **Ans.** | Thank you for your question. According to your suggestion, all figures are updated in the revised manuscript. |
| **Q9.** | Ethical and Data Source Clarification. The manuscript should clearly state whether ethical approval was required for the use of hospital data and how patient confidentiality was ensured, even if the data are aggregated. |
| **Ans.** | Thank you very much for the comment. The study used publicly available aggregated tuberculosis data from the WHO Global Tuberculosis Programme database (https://www.who.int/teams/global-tuberculosis-programme/data), and no direct human participation or individual consent was required; additionally, ethical approval was not required according to institutional guidelines. |

**Response to the Reviewer # 3**

| **Q1.** | Comment 1: Abstract Please consider/incorporate the followings points in the abstract i. aim/ objectives ii. Methods applied iii. Results/ Findings iv. Recommendations to policymakers |
| --- | --- |
| **Ans.** | Thank you and we appreciate your question. According to your opinion, the abstract has been reconstructed in the revised Manuscript. |
| **Q2.** | **Comment 2**: Introduction Please consider/incorporate the following points in the introduction. i. Significance of the research ii. Please clearly indicate in a summarized form your observations about the related literature reviewed as compared to your research. |
| **Ans.** | According to the suggestion, the introduction section has been modified to incorporate the above assumptions. [Section 1 at the end of page 2] |
| **Q3.** | **Comment 3:** Research gap and main contribution At the ending section of the introduction: i. Authors should clearly state the research gap in the manuscript. ii. SEIR/SEITR TB models with: treatment control, distancing or awareness control, PMP-based optimal control, sensitivity analysis have been extensively studied (including several papers already cited). Authors should compare their research with the existing literature and state what exactly is new in their research (main contribution) compared to existing studies. iii. The claimed novelty which comprises “composite analytical methodology integrating control-oriented stability decomposition with normalized forward sensitivity indexing” is not clearly demonstrated mathematically. Authors should check that. |
| **Ans.** | According to the suggestion, the introduction section has been modified to incorporate the above assumptions. [Section 1 at the end of page 2-3]  And, normalized forward sensitivity indexing is updated in **Section 6 (6.1)** and Table 1 on **page 13**. |
| **Q4.** | **Comment 4:** Stability analysis The stability analysis must be improved. Authors analyzed only DFE stability. Authors should consider Endemic equilibrium (EE) existence, Local or global stability of EE and Backward bifurcation analysis. Since TB models, normally exhibit backward bifurcation and highly relevant. |
| **Ans.** | Thank you very much for your suggestion. According to this, the stability analysis of DFE and EE is updated in the revised manuscript. [Subsection 4.1- 4.5] |
| **Q5.** | **Comment 5:** Sensitivity analysis, Authors conducted so many sensitivity plots, with little insight. Parameter ranges are not clearly justified and some interpretations are inconsistent (e.g., γ showing weak PRCC but strong analytical sensitivity). Authors should correct that. |
| **Ans.** | Thank you very much for such a comment. Parameter ranges are considered in Table 2 (Page 14), and the PRCC of γ is now correct in the revised manuscript. (Subsection 6.2 page 14). |
| **Q6.** | **Comment 6:** Grammatical Errors, There are some grammatical errors in the manuscript. For instance: 1.“Pontryagain’s” → Pontryagin’s 2. Repetitive phrases (“It is noted that…”, “It is observed that…”) and 3. Overly long descriptive captions instead of analytical discussion |
| **Ans.** | All grammatical issues have been corrected in the revised manuscript. |
| **Q7.** | **Comment 7**: Inconsistencies in Notations, 1. The authors used \lambda for both force of infection and adjoint derivative,2. Parameters appear in equations but are not always defined immediately3. Table 1 includes parameter \eta which does not appear in the model. Authors should correct that. |
| **Ans.** | According to the suggestions, all corrections have been considered in the revised manuscript. (page 10 and page 27) |
| **Q8.** | **Comment 9:** In Text-Reference, A lot of in-text references are missing in the manuscript. Authors should correct that. |
| **Ans.** | All missing citations have been fixed. |
| **Q9.** | **Comment 10:** Conclusion, State the limitations and future work in this section. |
| **Ans.** | According to the suggestion, the last paragraph of the conclusion section has been updated in the revised manuscript. (Section 10, page 23) |
| **Q10.** | **Comment 11:** Cost-effectiveness analysis, Cost-effectiveness analysis can be added to the control model to improve the manuscript. |
| **Ans.** | We appreciate this valuable suggestion. According to the suggestion, a new section is added in the revised manuscript. (Section 9) |
