# Supplementary material for: Dynamical behavior analysis of 2-control strategies on tuberculosis model
Source: PLOS Glob Public Health. 2026 Jun 8;6(6):e0005875. doi: 10.1371/journal.pgph.0005875 (PMC13245803; doi:10.1371/journal.pgph.0005875)
Supplement: Attachment — Submitted filename: Response to Reviewers_2nd.docx [file pgph.0005875.s011.docx]

**Response to the Reviewer**

**Title:** Dynamical Behavior Analysis of 2-control Strategies on Tuberculosis Model
**Paper ID: PGPH-D-26-00093**
PLOS Global Public Health.

**Dear Editor and Reviewers**

We sincerely thank the Editor and all Reviewers for their careful evaluation of our manuscript and for their insightful and constructive comments. We have thoroughly revised the manuscript in response to all suggestions. We believe these revisions have significantly strengthened the clarity, transparency, rigor, and overall quality of our work.

Below, we respond to each comment individually.

**Response to the Reviewer #2:**

| **Q1.** | The authors have adequately addressed all comments raised in the previous round of review. The revisions have significantly improved the clarity, methodological rigor, and overall presentation of the manuscript. The study presents a well-structured and technically sound analysis of the dynamical behavior of tuberculosis transmission under two control strategies. The modeling framework is appropriate, and the assumptions are clearly stated and justified. The statistical and mathematical analyses appear to be conducted rigorously, and the results are presented in a coherent and interpretable manner. Importantly, the conclusions are well supported by the data and align with the objectives of the study. The authors have also ensured compliance with data availability requirements, which enhances transparency and reproducibility. The manuscript is clearly written in standard English, with improved organization and readability compared to the previous version. Any minor typographical or grammatical issues noted earlier have been appropriately corrected. Overall, the manuscript now meets the publication criteria of PLOS Global Public Health and is suitable for publication. |
| --- | --- |
| **Ans.** | We are truly grateful to the Reviewer for the positive and encouraging evaluation of our revised manuscript. We appreciate your acknowledgement that our revisions have improved the clarity, methodological rigor, and presentation of our work. Furthermore, we are thankful for your comments confirming that the manuscript now meets publication standards. |

**Response to the Reviewer #3**

| **Q1.** | Specific: Focus on what is new (novel theory), the method, or data. |
| --- | --- |
| **Ans.** | Thank you for your insightful comment. The novelty of this work lies in developing a data-driven SEITR (Susceptible-Exposed-Infectious-Treated-Recovered) tuberculosis model that integrates two interacting control strategies—distancing and treatment—within a unified analytical framework. This framework is supported by a combination of global stability analysis, normalized sensitivity indices, and PRCC-based evaluation. Additionally, we present new findings by identifying threshold-driven dynamics and nonlinear interaction effects between the control strategies. These insights are original and relevant for policymakers aiming to optimize long-term tuberculosis intervention strategies. |
| **Q2.** | Employ Active Verbs: Begin them with phrases such as "We propose," "We discover," "We develop," or "We demonstrate" etc |
| **Ans.** | Thank you for this important suggestion. We have revised the manuscript to consistently use active voice, for example:   - We develop and rigorously analyze a deterministic SEITR (Susceptible–Exposed–Infectious–Treated–Recovered) model that incorporates distancing and treatment interventions to examine the transmission dynamics of tuberculosis. (**Abstract: page1 (line 1)**) - We propose a next-generation matrix formulation to determine the stability of the disease-free state and demonstrate that tuberculosis dies out whenever R_0_<1. (**Abstract: page1 (line 5)**)   These changes improve clarity and align with international scientific writing standards. |
| **Q3.** | The introduction should state the questions you asked, while the contribution section (or conclusion) should state the answers you found. |
| **Ans.** | **We sincerely thank the reviewer for this constructive suggestion. The manuscript has been revised so that the Introduction clearly states the research questions, while the Conclusion now explicitly summarizes the key findings, including the effectiveness of combined control strategies, the dominant role of the transmission rate (β), and the superior cost-effectiveness of dual interventions, thereby improving the overall logical flow and clarity.** |

**Response to the Reviewer # 4**

| **Q1.** | Authors need to conform to international guidelines of scientific writing. Sentences like "doctors typically diagnose..." can be better written with "healthcare professionals diagnose..." |
| --- | --- |
| **Ans.** | We sincerely thank the reviewer for the valuable suggestion, and the manuscript has been revised throughout to replace informal expressions with appropriate academic terminology in accordance with international scientific writing standards. (**Section 1: Introduction: page 2 (line 4)**) |
| **Q2.** | The introduction section has information about the basics of tuberculosis and models design that are not necessarily important to the study. Can revise and shorten. See comment above too. |
| **Ans.** | We appreciate this comment and have revised the Introduction to enhance clarity and focus. General background information on tuberculosis and repetitive descriptions of standard modeling approaches have been substantially reduced. Greater emphasis has been placed on the research gap, study motivation, and specific objectives. As a result, the section has been made more concise and directly aligned with the scope of the study. (**Section 1: Introduction: page 2 (line 31-37)**) |
| **Q3.** | In fact, it would be beneficial if you provide details about the current TB control strategy in Bangladesh and some background epidemiologic data about TB in Bangladesh (the country of interest) the introduction. |
| **Ans.** | We appreciate the reviewer's valuable suggestion and have enhanced the Introduction accordingly. We have included Bangladesh-specific epidemiological data, supported by validation data from the WHO, to emphasize the country's TB burden. The WHO-based data is utilized for numerical simulations and cost-effectiveness analysis in the revised manuscript. |
| **Q4.** | tuberculin skin test is use to diagnose TB "infection"- clarify this to readers |
| **Ans.** | We sincerely thank the reviewer for emphasizing this important point. The manuscript has been revised to clearly state that the tuberculin skin test (TST) is used for diagnosing latent tuberculosis infection rather than active disease, thereby improving accuracy and avoiding ambiguity. (**Section 1: Introduction_page 2(line 4-5)** ) |
| **Q5.** | I assume that the first control strategy "treatment" involves treatment of active TB disease, not latent infection. |
| **Ans.** | We appreciate the reviewer for their insightful and correct observation. In the revised manuscript, we clarify that the treatment control (u_2_) specifically refers to the treatment of individuals with active infectious tuberculosis (TB) as they transition from the infectious compartment (I) to the treated compartment (T). This ensures consistency with the model structure and its epidemiological interpretation. |
| **Q6.** | Not sure if authors accounted for failure of TB treatment in the model? |
| **Ans.** | We appreciate the reviewer's concern and would like to clarify that our current model assumes that treatment results in either recovery or disease progression, without explicitly accounting for treatment failure. Instead, we use treatment-related parameters (γ, τ, δ_1_) to indirectly represent outcomes such as recovery and mortality caused by the disease. The limitations about treatment are acknowledged in the last part of conclusion in the revised manuscript. (**Section 10: Conclusions: page 24_last paragraph**) |
| **Q7.** | What is the prevalence of MDR-TB in Bangladesh and was this accounted for in the transmission model and cost-effectiveness model? |
| **Ans.** | We appreciate this important observation. The current model does not include multidrug-resistant tuberculosis (MDR-TB) as a separate compartment. However, its possible effects are partly captured through treatment effectiveness (τ) and disease-induced mortality (δ_1_). An explicitly modeling MDR-TB would require an extension of the current model and will be considered in the future research. |

**Report of Figure Resubmissions:**

Dear editor, Thank you for your patience and understanding. I regret that I was unable to use the NAAS tool for converting the figures included in the revised manuscript; however, to ensure compliance with the journal requirements, I have attached all figures separately in both EPS and TIFF formats for your convenience, and I sincerely apologize for any inconvenience caused.

**Final Statement**

We sincerely thank the Reviewer and Editor for their valuable feedback and constructive suggestions. These comments have significantly improved the manuscript in terms of methodological rigor, mathematical clarity, data transparency, reproducibility, language quality and relevance of our manuscript. We respectfully hope that the revised version meets the standards of PLOS Global Public Health and is now suitable for publication and we respectfully submit it for reconsideration.
