# Supplementary material for: Dynamical behavior analysis of 2-control strategies on tuberculosis model
Source: PLOS Glob Public Health. 2026 Jun 8;6(6):e0005875. doi: 10.1371/journal.pgph.0005875 (PMC13245803; doi:10.1371/journal.pgph.0005875)
Supplement: Peer review history

# Peer review: Decision Letter 0

**DOI:** 10.1371/journal.pgph.0005875.r001

**Authors:** Somayeh Hessam

2 Feb 2026

PGPH-D-26-00093

Dynamical Behavior Analysis of 2-control Strategies on Tuberculosis Model

PLOS Global Public Health

Dear Dr. Nayeem,

Thank you for submitting your manuscript to PLOS Global Public Health. After careful consideration, we feel that it has merit but does not fully meet PLOS Global Public Health’s publication criteria as it currently stands. Therefore, we invite you to submit a revised version of the manuscript that addresses the points raised during the review process.

Please submit your revised manuscript by Mar 19 2026 11:59PM. If you will need more time than this to complete your revisions, please reply to this message or contact the journal office at globalpubhealth@plos.org. When you're ready to submit your revision, log on to https://www.editorialmanager.com/pgph/ and select the 'Submissions Needing Revision' folder to locate your manuscript file.

Please include the following items when submitting your revised manuscript:

- A letter that responds to each point raised by the editor and reviewer(s). You should upload this letter as a separate file labeled 'Response to Reviewers'.
- A marked-up copy of your manuscript that highlights changes made to the original version. You should upload this as a separate file labeled 'Revised Manuscript with Track Changes'.
- An unmarked version of your revised paper without tracked changes. You should upload this as a separate file labeled 'Manuscript'.

Guidelines for resubmitting your figure files are available below the reviewer comments at the end of this letter.

We look forward to receiving your revised manuscript.

Kind regards,

Somayeh Hessam

Academic Editor

PLOS Global Public Health

Journal Requirements:

1. Please note that PLOS Global Public Health has specific guidelines on code sharing for submissions in which author-generated code underpins the findings in the manuscript. In these cases, all author-generated code must be made available without restrictions upon publication of the work. Please review our guidelines at https://journals.plos.org/globalpublichealth/s/materials-and-software-sharing#loc-sharing-code and ensure that your code is shared in a way that follows best practice and facilitates reproducibility and reuse.

2. In the ethics statement in the Methods, you have specified that verbal consent was obtained. Please provide additional details regarding how this consent was documented and witnessed, and state whether this was approved by the IRB.

3. Please provide a detailed online Financial Disclosure statement. This is published with the article. It must therefore be completed in full sentences and contain the exact wording you wish to be published.

a) State the initials, alongside each funding source, of each author to receive each grant. For example: “This work was supported by the National Institutes of Health (####### to AM; ###### to CJ) and the National Science Foundation (###### to AM).”

b) State what role the funders took in the study. If the funders had no role in your study, please state: “The funders had no role in study design, data collection and analysis, decision to publish, or preparation of the manuscript.”

For more information, please go to our submission guidelines:

https://journals.plos.org/globalpublichealth/s/submission-guidelines#loc-financial-disclosure-statement

4. Please ensure that the funders and grant numbers match between the Financial Disclosure field and the Funding Information tab in your submission form. Note that the funders must be provided in the same order in both places as well.

5. Please update your online Competing Interests statement. If you have no competing interests to declare, please state: “The authors have declared that no competing interests exist.”

6. Thank you for uploading your study's underlying data set. Unfortunately, the repository you have noted in your Data Availability statement does not qualify as an acceptable data repository according to PLOS's standards.

At this time, please upload the minimal data set necessary to replicate your study's findings to a stable, public repository (such as figshare or Dryad) and provide us with the relevant URLs, DOIs, or accession numbers that may be used to access these data. For a list of recommended repositories and additional information on PLOS standards for data deposition, please see https://journals.plos.org/globalpublichealth/s/recommended-repositories.

7. We note that you may not have used the PLOS LaTeX template. This is not required, but please be sure that you are familiar with our LaTeX guidelines to help reduce the chance of delay due to typesetting errors. Our guidelines can be found here: https://journals.plos.org/digitalhealth/s/latex

8. Please ensure that you have provided a single, cohesive .tex file for your LaTeX submission. As stated in the PLOS template, your references should be included in your .tex file (not submitted separately as .bib or .bbl). Please also ensure that you are making any formatting changes to both your .tex file and the PDF of your manuscript. If you have any questions, please contact Latex@plos.org. You can find our LaTeX guidelines here: https://journals.plos.org/digitalhealth/s/latex

9. Please provide separate main figure files in .tif or .eps format only and ensure that all files are under our size limit of 10MB. You may leave the embedded figures in the manuscript.

For more information about how to convert your figure files please see our guidelines: https://journals.plos.org/globalpublichealth/s/figures

10. We notice that your supplementary material (Appendix) is included in the manuscript file. Please remove them and upload them with the file type 'Supporting Information'. Please ensure that each Supporting Information file has a legend listed in the manuscript before or after the references list.

11. If the reviewer comments include a recommendation to cite specific previously published works, please review and evaluate these publications to determine whether they are relevant and should be cited. There is no requirement to cite these works unless the editor has indicated otherwise.

Additional Editor Comments (if provided):

[Note: HTML markup is below. Please do not edit.]

Reviewers' comments:

Reviewer's Responses to Questions

**Comments to the Author**

1. Does this manuscript meet PLOS Global Public Health’s publication criteria? Is the manuscript technically sound, and do the data support the conclusions? The manuscript must describe methodologically and ethically rigorous research with conclusions that are appropriately drawn based on the data presented.

Reviewer #1: Partly

Reviewer #2: Partly

Reviewer #3: Yes

**********

2. Has the statistical analysis been performed appropriately and rigorously?

Reviewer #1: Yes

Reviewer #2: No

Reviewer #3: Yes

**********

3. Have the authors made all data underlying the findings in their manuscript fully available (please refer to the Data Availability Statement at the start of the manuscript PDF file)?

The PLOS Data policy requires authors to make all data underlying the findings described in their manuscript fully available without restriction, with rare exception. The data should be provided as part of the manuscript or its supporting information, or deposited to a public repository. For example, in addition to summary statistics, the data points behind means, medians and variance measures should be available. If there are restrictions on publicly sharing data—e.g. participant privacy or use of data from a third party—those must be specified.

Reviewer #1: No

Reviewer #2: No

Reviewer #3: No

**********

4. Is the manuscript presented in an intelligible fashion and written in standard English?

PLOS Global Public Health does not copyedit accepted manuscripts, so the language in submitted articles must be clear, correct, and unambiguous. Any typographical or grammatical errors should be corrected at revision, so please note any specific errors here.

Reviewer #1: No

Reviewer #2: No

Reviewer #3: Yes

**********

5. Review Comments to the Author

Please use the space provided to explain your answers to the questions above. You may also include additional comments for the author, including concerns about dual publication, research ethics, or publication ethics. (Please upload your review as an attachment if it exceeds 20,000 characters)

Reviewer #1: Recommendation 1: The modeling approach is fundamentally sound and relevant to TB control (a major public health issue), but the manuscript needs to address transparency (data sources, parameter justification) and clarify some methodological assumptions (e.g. definition of Reproduction number) before it can be accepted.

Recommendation 2: The sensitivity analysis methods (Pearson, PRCC) are suitable but need more rigor in reporting (sample details, uncertainty). The data-fitting procedure requires complete description (data source, fitting algorithm, goodness-of-fit). The authors should include more quantitative statistical assessment of their model fit and uncertainty in parameter effects.

Recommendation 3: The authors should provide a Data Availability Statement with actual access information (URL, DOI, or supplementary files) for the epidemiological data and any other material used. At the end of the file, an editorial link points to the Bangladesh NTP Annual Report 2022, which presumably provided TB case counts, but this is not an open dataset citation. Without accessible data, readers cannot reproduce or verify the results.

Recommendation 4: The writing requires major language editing. Grammatical issues and awkward phrasing occur repeatedly. Several citations are incomplete (marked as “[?]”). These issues hinder readability. The manuscript would benefit from careful editing by a fluent English speaker. With editing, the intellectual content is conveyable, but in its current state the language detracts from clarity.

Reviewer #2: This manuscript presents a mathematical modeling study on tuberculosis transmission using an SEITR framework incorporating distancing and treatment control strategies. The authors apply optimal control theory, stability analysis, sensitivity analysis, and data fitting to assess the impact of interventions. The topic is relevant to public health and epidemiological modeling, and the integration of control theory with real data is a valuable contribution. Overall, the study demonstrates potential scientific merit; however, several important issues should be addressed to improve the quality, clarity, and reliability of the work.

Major Strengths

The manuscript develops a structured SEITR model that includes exposed and treated compartments, allowing for a more realistic representation of tuberculosis dynamics.

The application of Pontryagin’s Maximum Principle to determine optimal intervention strategies is appropriate and well motivated.

The use of sensitivity analysis, including Pearson correlation and PRCC, strengthens the interpretation of key parameters.

Incorporation of real tuberculosis data from Bangladesh and curve fitting enhances the practical relevance of the study.

The findings highlight the importance of combined distancing and treatment strategies, which is relevant for policy planning.

Major Concerns

Language and Presentation

The manuscript contains numerous grammatical errors, awkward phrasing, and unclear sentences that affect readability. Several sections require substantial English language editing. Professional proofreading is strongly recommended to improve clarity and coherence.

Incomplete and Missing Citations

Many references in the text are marked with “[?]”, indicating missing citations. These must be replaced with appropriate and verifiable references. Failure to do so weakens the scientific credibility of the manuscript.

Reference Quality and Formatting

Several references appear incomplete, inconsistently formatted, or potentially inaccurate. All references should be verified and formatted according to journal guidelines. The authors should ensure that all cited sources are reliable and accessible.

Mathematical Notation and Consistency

Some notations are used ambiguously, particularly the symbol λ, which represents both the force of infection and adjoint variables. This may confuse readers. The notation should be revised for clarity. In addition, some derivations in the optimal control section are not sufficiently detailed and require clearer explanation.

Data Availability and Reproducibility

The Data Availability Statement is insufficient. PLOS requires full access to underlying data and, where applicable, code. The authors should provide the dataset in an accessible repository and include links. Details on parameter estimation and model fitting procedures should also be expanded.

Model Validation

While curve fitting is presented, quantitative goodness-of-fit measures (e.g., RMSE, R², confidence intervals) are not provided. Including these metrics would strengthen the validation of the model.

Overstatement of Novelty

The novelty section contains strong claims that are not fully supported by the analysis. The authors should moderate these statements and clearly specify how their approach differs from and extends existing studies.

Figures and Visualization

Some figures lack sufficient resolution, clear legends, and readable labels. All figures should be revised to meet publication standards and to improve interpretability.

Ethical and Data Source Clarification

The manuscript should clearly state whether ethical approval was required for the use of hospital data and how patient confidentiality was ensured, even if the data are aggregated.

Minor Concerns

Several typographical errors and formatting inconsistencies are present throughout the manuscript.

Some sections are repetitive, particularly in the Introduction and Discussion.

Units and parameter descriptions in the appendix should be clarified and consistently presented.

The structure of some tables can be improved for better readability.

Suggestions for Improvement

Revise the entire manuscript for language quality and clarity.

Replace all missing citations and verify references.

Improve mathematical notation and provide clearer derivations.

Expand the methodology for parameter estimation and numerical implementation.

Provide full data and code access in line with PLOS policies.

Include quantitative validation metrics.

Revise figures and tables for better presentation.

Reframe the novelty claims in a more balanced manner.

Conclusion

The manuscript addresses an important topic and combines mathematical modeling with real data and optimal control analysis. With substantial revision focusing on language quality, citation completeness, data transparency, mathematical clarity, and methodological rigor, the study has the potential to make a meaningful contribution to tuberculosis control research. At present, major revisions are necessary before the manuscript can be considered for publication.

Reviewer #3: PLOS Global Public Health (PGPH-D-26-00093)

Reviewer’s Comments

The authors studied ‘Dynamical Behavior Analysis of 2-control Strategies on Tuberculosis Model. The authors must be commended for carrying out this research. As the paper is, technically correct formulation of the SEITR system, Standard application of Pontryagin’s Maximum Principle, Inclusion of sensitivity analysis and data fitting and clear policy motivation (treatment and distancing synergy). However, authors need to address the following suggestions to improve the manuscript:

Comment 1: Abstract

Please consider/incorporate the followings points in the abstract

i. aim/ objectives

ii. Methods applied

iii. Results/ Findings

iv. Recommendations to policymakers

Comment 2: Introduction

Please consider/incorporate the following points in the introduction.

i. Significance of the research

ii. Please clearly indicate in a summarized form your observations about the related literature reviewed as compared to your research.

Comment 3: Research gap and main contribution

At the ending section of the introduction:

i. Authors should clearly state the research gap in the manuscript.

ii. SEIR/SEITR TB models with: treatment control, distancing or awareness control, PMP-based optimal control, sensitivity analysis have been extensively studied (including several papers already cited). Authors should compare their research with the existing literature and state what exactly is new in their research (main contribution) compared to existing studies.

iii. The claimed novelty which comprises “composite analytical methodology integrating control-oriented stability decomposition with normalized forward sensitivity indexing” is not clearly demonstrated mathematically. Authors should check that.

Comment 4: Stability analysis

The stability analysis must be improved. Authors analyzed only DFE stability. Authors should consider Endemic equilibrium (EE) existence, Local or global stability of EE and Backward bifurcation analysis. Since TB models, normally exhibit backward bifurcation and highly relevant.

Comment 5: Sensitivity analysis

Authors conducted so many sensitivity plots, with little insight. Parameter ranges are not clearly justified and some interpretations are inconsistent (e.g., γ showing weak PRCC but strong analytical sensitivity). Authors should correct that.

Comment 6: Grammatical Errors

There are some grammatical errors in the manuscript. For instance

1. “Pontryagain’s” → Pontryagin’s

2. Repetitive phrases (“It is noted that…”, “It is observed that…”) and

3. Overly long descriptive captions instead of analytical discussion

Comment 7: Inconsistencies in Notations

1. The authors used \lambda for both force of infection and adjoint derivative,

2. Parameters appear in equations but are not always defined immediately

3. Table 1 includes parameter \eta which does not appear in the model

Authors should correct that.

Comment 9: In Text-Reference

A lot of in-text references are missing in the manuscript. Authors should correct that.

Comment 10: Conclusion

State the limitations and future work in this section.

Comment 11: Cost-effectiveness analysis

Cost-effectiveness analysis can be added to the control model to improve the manuscript.

Remarks: Minor Revision Required

Thank you

**********

6. PLOS authors have the option to publish the peer review history of their article (what does this mean?). If published, this will include your full peer review and any attached files.

**Do you want your identity to be public for this peer review?** If you choose “no”, your identity will remain anonymous but your review may still be made public.

For information about this choice, including consent withdrawal, please see our Privacy Policy.

Reviewer #1: **Yes:** Uzair Ahmed

Reviewer #2: No

Reviewer #3: No

**********

[NOTE: If reviewer comments were submitted as an attachment file, they will be attached to this email and accessible via the submission site. Please log into your account, locate the manuscript record, and check for the action link "View Attachments". If this link does not appear, there are no attachment files.]

-->Figure Resubmissions:

While revising your submission, we strongly recommend that you use PLOS’s NAAS tool (https://ngplosjournals.pagemajik.ai/artanalysis) to test your figure files. NAAS can convert your figure files to the TIFF file type and meet basic requirements (such as print size, resolution), or provide you with a report on issues that do not meet our requirements and that NAAS cannot fix.

After uploading your figures to PLOS’s NAAS tool - https://ngplosjournals.pagemajik.ai/artanalysis, NAAS will process the files provided and display the results in the "Uploaded Files" section of the page as the processing is complete. If the uploaded figures meet our requirements (or NAAS is able to fix the files to meet our requirements), the figure will be marked as "fixed" above. If NAAS is unable to fix the files, a red "failed" label will appear above. When NAAS has confirmed that the figure files meet our requirements, please download the file via the download option, and include these NAAS processed figure files when submitting your revised manuscript.

Attachment Submitted filename: Review Comments for PLOS HEALTH REVIEW.docx

# Author comment: Author response to Decision Letter 1

**DOI:** 10.1371/journal.pgph.0005875.r002

8 Mar 2026

Attachment Submitted filename: Response to Reviewers.docx

# Peer review: Decision Letter 1

**DOI:** 10.1371/journal.pgph.0005875.r003

**Authors:** Somayeh Hessam

12 Apr 2026

PGPH-D-26-00093R1

Dynamical Behavior Analysis of 2-control Strategies on Tuberculosis Model

PLOS Global Public Health

Dear Dr. Nayeem,

Thank you for submitting your manuscript to PLOS Global Public Health. After careful consideration, we feel that it has merit but does not fully meet PLOS Global Public Health’s publication criteria as it currently stands. Therefore, we invite you to submit a revised version of the manuscript that addresses the points raised during the review process.

Please submit your revised manuscript by May 12 2026 11:59PM. If you will need more time than this to complete your revisions, please reply to this message or contact the journal office at globalpubhealth@plos.org. When you're ready to submit your revision, log on to https://www.editorialmanager.com/pgph/ and select the 'Submissions Needing Revision' folder to locate your manuscript file.

Please include the following items when submitting your revised manuscript:

- A letter that responds to each point raised by the editor and reviewer(s). You should upload this letter as a separate file labeled 'Response to Reviewers'.
- A marked-up copy of your manuscript that highlights changes made to the original version. You should upload this as a separate file labeled 'Revised Manuscript with Track Changes'.
- An unmarked version of your revised paper without tracked changes. You should upload this as a separate file labeled 'Manuscript'.

Guidelines for resubmitting your figure files are available below the reviewer comments at the end of this letter.

As the corresponding author, your ORCID iD is verified in the submission system and will appear in the published article. PLOS supports the use of ORCID, and we encourage all coauthors to register for an ORCID iD and use it as well. Please encourage your coauthors to verify their ORCID iD within the submission system before final acceptance, as unverified ORCID iDs will not appear in the published article. *Only* the individual author can complete the verification step; PLOS staff *cannot* verify ORCID iDs on behalf of authors.

We look forward to receiving your revised manuscript.

Kind regards,

Somayeh Hessam

Academic Editor

PLOS Global Public Health

**Journal Requirements:**

If the reviewer comments include a recommendation to cite specific previously published works, please review and evaluate these publications to determine whether they are relevant and should be cited. There is no requirement to cite these works unless the editor has indicated otherwise.

Please review your reference list to ensure that it is complete and correct. If you have cited papers that have been retracted, please include the rationale for doing so in the manuscript text, or remove these references and replace them with relevant current references. Any changes to the reference list should be mentioned in the rebuttal letter that accompanies your revised manuscript. If you need to cite a retracted article, indicate the article’s retracted status in the References list and also include a citation and full reference for the retraction notice.

**Additional Editor Comments (if provided):**

[Note: HTML markup is below. Please do not edit.]

Reviewers' comments:

Reviewer's Responses to Questions

**Comments to the Author**

1. If the authors have adequately addressed your comments raised in a previous round of review and you feel that this manuscript is now acceptable for publication, you may indicate that here to bypass the “Comments to the Author” section, enter your conflict of interest statement in the “Confidential to Editor” section, and submit your "Accept" recommendation.

Reviewer #2: All comments have been addressed

Reviewer #3: All comments have been addressed

Reviewer #4: All comments have been addressed

**********

2. Does this manuscript meet PLOS Global Public Health’s publication criteria? Is the manuscript technically sound, and do the data support the conclusions? The manuscript must describe methodologically and ethically rigorous research with conclusions that are appropriately drawn based on the data presented.

Reviewer #2: Yes

Reviewer #3: Yes

Reviewer #4: Yes

**********

3. Has the statistical analysis been performed appropriately and rigorously?

Reviewer #2: Yes

Reviewer #3: Yes

Reviewer #4: Yes

**********

4. Have the authors made all data underlying the findings in their manuscript fully available (please refer to the Data Availability Statement at the start of the manuscript PDF file)?

The PLOS Data policy requires authors to make all data underlying the findings described in their manuscript fully available without restriction, with rare exception. The data should be provided as part of the manuscript or its supporting information, or deposited to a public repository. For example, in addition to summary statistics, the data points behind means, medians and variance measures should be available. If there are restrictions on publicly sharing data—e.g. participant privacy or use of data from a third party—those must be specified.

Reviewer #2: Yes

Reviewer #3: Yes

Reviewer #4: Yes

**********

5. Is the manuscript presented in an intelligible fashion and written in standard English?

PLOS Global Public Health does not copyedit accepted manuscripts, so the language in submitted articles must be clear, correct, and unambiguous. Any typographical or grammatical errors should be corrected at revision, so please note any specific errors here.

Reviewer #2: Yes

Reviewer #3: Yes

Reviewer #4: Yes

**********

6. Review Comments to the Author

Please use the space provided to explain your answers to the questions above. You may also include additional comments for the author, including concerns about dual publication, research ethics, or publication ethics. (Please upload your review as an attachment if it exceeds 20,000 characters)

**Reviewer #2:** The authors have adequately addressed all comments raised in the previous round of review. The revisions have significantly improved the clarity, methodological rigor, and overall presentation of the manuscript.

The study presents a well-structured and technically sound analysis of the dynamical behavior of tuberculosis transmission under two control strategies. The modeling framework is appropriate, and the assumptions are clearly stated and justified. The statistical and mathematical analyses appear to be conducted rigorously, and the results are presented in a coherent and interpretable manner.

Importantly, the conclusions are well supported by the data and align with the objectives of the study. The authors have also ensured compliance with data availability requirements, which enhances transparency and reproducibility.

The manuscript is clearly written in standard English, with improved organization and readability compared to the previous version. Any minor typographical or grammatical issues noted earlier have been appropriately corrected.

Overall, the manuscript now meets the publication criteria of PLOS Global Public Health and is suitable for publication.

**Reviewer #3:** Dear Editor

PLOS Global Public Health(PGPH-D-26-00093R1)

Reviewer’s Comments

The authors studied ‘Dynamical Behavior Analysis of 2-control Strategies on Tuberculosis Model. I have checked the revised version of the manuscript according to the comments given. The authors have revised the manuscript according to the comments. I suggest that the manuscript can be accepted for publication PLOS Global Public Health after this minor suggestion. However, authors need to address the suggestion below to improve the manuscript:

Comment on main Contribution

The main contributions must not be stated as questions. Authors should correct that. Authors should be:

1. Specific: Focus on what is new (novel theory), the method, or data.

2. Employ Active Verbs: Begin them with phrases such as "We propose," "We discover," "We develop," or "We demonstrate" etc

3. The introduction should state the questions you asked, while the contribution section

(or conclusion) should state the answers you found.

Remarks: Accept

Thank you

**Reviewer #4:** Interesting study, here are my recommendations to improve the manuscript:

- Authors need to conform to international guidelines of scientific writing. Sentences like "doctors typically diagnose..." can be better written with "healthcare professionals diagnose..."

- The introduction section has information about the basics of tuberculosis and models design that are not necessarily important to the study. Can revise and shorten. See comment above too.

- In fact, it would be beneficial if you provide details about the current TB control strategy in Bangladesh and some background epidemiologic data about TB in Bangladesh (the country of interest) the introduction.

- tuberculin skin test is use to diagnose TB "infection"- clarify this to readers

- I assume that the first control strategy "treatment" involves treatment of active TB disease, not latent infection.

- Not sure if authors accounted for failure of TB treatment in the model?

- What is the prevalence of MDR-TB in Bangladesh and was this accounted for in the transmission model and cost-effectiveness model?

**********

7. PLOS authors have the option to publish the peer review history of their article (what does this mean?). If published, this will include your full peer review and any attached files.

**Do you want your identity to be public for this peer review?** If you choose “no”, your identity will remain anonymous but your review may still be made public.

For information about this choice, including consent withdrawal, please see our Privacy Policy.

Reviewer #2: **Yes:** ABDULRAZAK MOHAMED AHMED QUDDE

Reviewer #3: No

Reviewer #4: No

**********

[NOTE: If reviewer comments were submitted as an attachment file, they will be attached to this email and accessible via the submission site. Please log into your account, locate the manuscript record, and check for the action link "View Attachments". If this link does not appear, there are no attachment files.]

**Figure Resubmissions:**

While revising your submission, we strongly recommend that you use PLOS’s NAAS tool (https://ngplosjournals.pagemajik.ai/artanalysis) to test your figure files. NAAS can convert your figure files to the TIFF file type and meet basic requirements (such as print size, resolution), or provide you with a report on issues that do not meet our requirements and that NAAS cannot fix.

After uploading your figures to PLOS’s NAAS tool - https://ngplosjournals.pagemajik.ai/artanalysis, NAAS will process the files provided and display the results in the "Uploaded Files" section of the page as the processing is complete. If the uploaded figures meet our requirements (or NAAS is able to fix the files to meet our requirements), the figure will be marked as "fixed" above. If NAAS is unable to fix the files, a red "failed" label will appear above. When NAAS has confirmed that the figure files meet our requirements, please download the file via the download option, and include these NAAS processed figure files when submitting your revised manuscript.

# Author comment: Author response to Decision Letter 2

**DOI:** 10.1371/journal.pgph.0005875.r004

23 Apr 2026

Attachment Submitted filename: Response to Reviewers_2nd.docx

# Editor report: Decision Letter 2

**DOI:** 10.1371/journal.pgph.0005875.r005

**Authors:** Somayeh Hessam

26 Apr 2026

Dynamical Behavior Analysis of 2-control Strategies on Tuberculosis Model

PGPH-D-26-00093R2

Dear Dr. Nayeem,

We are pleased to inform you that your manuscript 'Dynamical Behavior Analysis of 2-control Strategies on Tuberculosis Model' has been provisionally accepted for publication in PLOS Global Public Health.

Before your manuscript can be formally accepted you will need to complete some formatting changes, which you will receive in a follow up email. A member of our team will be in touch with a set of requests.

Please note that your manuscript will not be scheduled for publication until you have made the required changes, so a swift response is appreciated.

IMPORTANT: The editorial review process is now complete. PLOS will only permit corrections to spelling, formatting or significant scientific errors from this point onwards. Requests for major changes, or any which affect the scientific understanding of your work, will cause delays to the publication date of your manuscript.

If your institution or institutions have a press office, please notify them about your upcoming paper to help maximize its impact. If they'll be preparing press materials, please inform our press team as soon as possible -- no later than 48 hours after receiving the formal acceptance. Your manuscript will remain under strict press embargo until 2 pm Eastern Time on the date of publication. For more information, please contact globalpubhealth@plos.org.

Thank you again for supporting Open Access publishing; we are looking forward to publishing your work in PLOS Global Public Health.

Best regards,

Somayeh Hessam

Academic Editor

PLOS Global Public Health

***********************************************************

Reviewer Comments (if any, and for reference):
